# Supplementary material for: Nucleation-promoting and growth-limiting synthesis of disordered rock-salt Li-ion cathode materials
Source: Nat Commun. 2025 Jul 1;16:5806. doi: 10.1038/s41467-025-60946-4 (PMC12216312; doi:10.1038/s41467-025-60946-4)
Supplement: Supplementary file 1 — Supplementary Information [file 41467_2025_60946_MOESM1_ESM.pdf]

Supplementary Information

Nucleation-promoting and growth-limiting synthesis of disordered rock-salt Li-ion cathode materials

Hoda Ahmed[1], Moohyun Woo[1], Nicolas Dumaresq[1], Pablo Trevino Lara[1], Richie Fong[1], Sang-Jun Lee[2], Gregory Lazaris[1], Nauman Mubarak[1], Nicolas Brodusch[1], Dong-Hwa Seo[3], Raynald Gauvin[1], George P. Demopoulos[1], and Jinhyuk Lee[1]*

[1] Department of Mining and Materials Engineering, McGill University, Montreal, QC H3A 0C5, Canada

[2] Stanford Synchrotron Radiation Lightsource, SLAC National Accelerator Laboratory, Menlo Park, CA, 94025, USA

[3] Department of Materials Science and Engineering, Korea Advanced Institute of Science and Technology (KAIST), 291 Daehak-ro, Daejeon 34141, Republic of Korea

*Correspondence and requests for materials should be addressed to Jinhyuk Lee (jinhyuk.lee@mcgill.ca)

Table of contents

- Supplementary Figure 1 | Salt-flux effect using Cs/K halides (XRD, SEM, salt properties).
- Supplementary Figure 2 | Effect of precursor-to-salt weight ratio (XRD, SEM).
- Supplementary Figure 3 | XRD refinements of S-LMTO and PS-LMTO.
- Supplementary Figure 4 | XRD refinements of NM-LMTO at varying calcination temps/times.
- Supplementary Figure 5 | SEM + particle size distribution for S-/PS-/NM-LMTO.
- Supplementary Figure 6 | XRD refinements of NM-LMTO-70, NM-LMNO, NM-LNTO; ICP-OES.
- Supplementary Figure 7 | SEM + particle size distribution of NM-LMTO-70, NM-LMNO, NM-LNTO.
- Supplementary Figure 8 | XRD refinement of NM-LMTO without annealing.
- Supplementary Figure 9 | XRD refinements of NM-LMTO with different annealing times.
- Supplementary Figure 10 | pH titration of wash solution (LiOH, Li_2CO_3 quantification).

- Supplementary Figure 11 | ICP-OES Li content recovery vs. pH of washing; electrochemical profiles (pH 7–14).
- Supplementary Figure 12 | Li||NM/PS-LMTO voltage, capacity retention, CE for PS- and NM-LMTO using different carbon additives.
- Supplementary Figure 13 | Same as above with mixing and carbon type varied.
- Supplementary Figure 14 | NM-LMTO (annealed at 600°C-10h) in optimized 70:20:10 MWCNT electrode.
- Supplementary Figure 15 | Graphite anode profile in a Li||Graphite cell, Graphite||NM/PS-LMTO cell performance.
- Supplementary Note 1 | Graphite||NM/PS-LMTO cell performance and capacity fade interpretation.
- Supplementary Figure 16 | Li||PS-LMTO cell at lower voltage window (1.5–4.4 V).
- Supplementary Figure 17 | NM-LMTO electrode films: before/after 40 and 100 cycles (XRD).
- Supplementary Figure 18 | PS-LMTO electrode films: before/after 40 and 100 cycles (XRD).
- Supplementary Figure 19 | Mn L_3-edge and O *K*-edge XAS (Pristine, Charged, Discharged).
- Supplementary Note 2 | Redox dynamics, Mn^{2+} formation, oxygen loss comparison.
- Supplementary Figure 20 | O 1*s* XPS: CEI formation comparison in NM vs PS-LMTO.
- Supplementary Figure 21 | Mn deposition on Li-metal anode (ICP-OES).
- Supplementary Figure 22 | Equivalent circuit and Nyquist plots of Li‖NM/PS-LMTO cells before and after 100 cycles (1.5–4.8 V, 20 mA/g, 25 °C).
- Supplementary Table 1 | Fitted EIS parameters with standard errors corresponding to the Nyquist plots in Supplementary Fig. 22.
- Supplementary Figure 23 | Equivalent circuit and Nyquist plots of NM/PS-LMTO‖ NM/PS-LMTO and Li‖Li symmetric cells constructed from Li‖NM/PS-LMTO cells before cycling and after 10 cycles (1.5–4.8 V, 20 mA/g, 25 °C).
- Supplementary Table 2 | Fitted EIS parameters with standard errors corresponding to the Nyquist plots in Supplementary Fig. 23.
- Supplementary Figure 24 | Equivalent circuit and Nyquist plots of three-electrode cells (NM/PS-LMTO as cathode, Li-deposited Cu mesh as reference, and Li-metal chip as counter electrode) before cycling and after 10 cycles (1.5–4.8 V, 20 mA/g, 25 °C)
- Supplementary Table 3 | Fitted EIS parameters with standard errors corresponding to the Nyquist plots in Supplementary Fig. 24.
- Supplementary Note 3 | Cell impedance analysis.

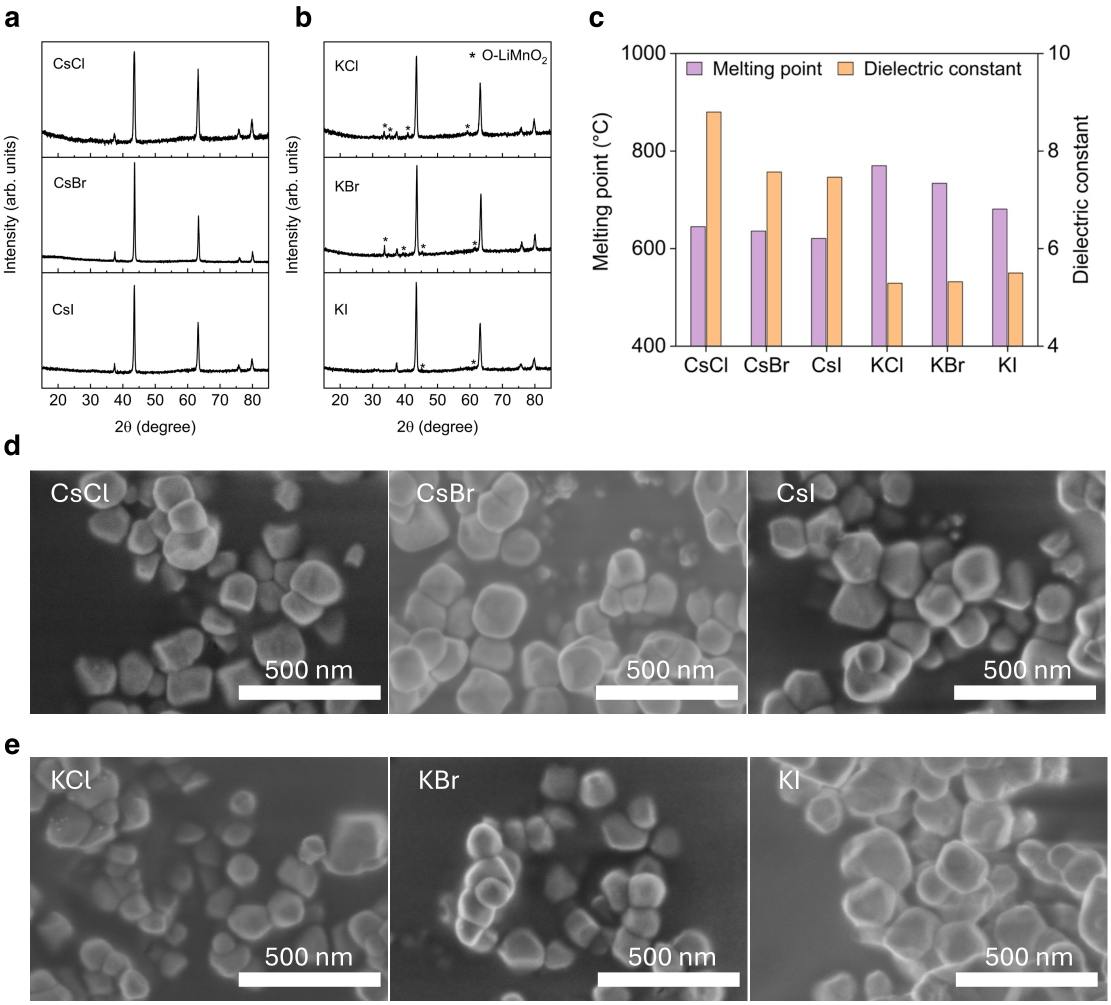

Supplementary Figure 1 | The salt-flux effect on the NM synthesis of LMTO (calcined at 800°C for 5 minutes and annealed at 600°C for 20 hours with the precursor-to-salt weight ratio of 1:1.5, followed by DIW washing and Li-reinsertion). (**a, b**) XRD patterns of the NM-LMTO synthesized using **a** cesium salts (CsCl, CsBr, and CsI) and **b** potassium salts (KCl, KBr, and KI), respectively. **c** melting points and dielectric constants of different cesium and potassium salts. (**d, e**) SEM images of the NM-LMTO made with **d** cesium salts (CsCl, CsBr, and CsI) and **e** potassium salts (KCl, KBr, and KI), respectively. Unlike the cesium salts, which lead to a pure LMTO phase, the potassium salts result in the impurity $LiMnO_2$ phase in the sample, indicating incomplete LMTO formation. This is likely due to the higher melting points and lower dielectric constants of the potassium salts, which limit the dissolution of LMTO precursors into the solvents during the short 800°C calcination step. Note that molten salts with a high dielectric constant generally enhance the solubility of ionic or polar precursors, supporting more effective synthesis reactions by providing a more stable and dissociative medium for the precursors. This enhanced molten-salt synthesis, in turn, allows the DRX synthesis to complete faster under the short molten-salt calcination time (*e.g.*, 5 min) in our experiments. The NM synthesis with different salt fluxes all results in sub-200 nm LMTO particles. Source data for Figs. S1a–c are provided as a Source Data file.

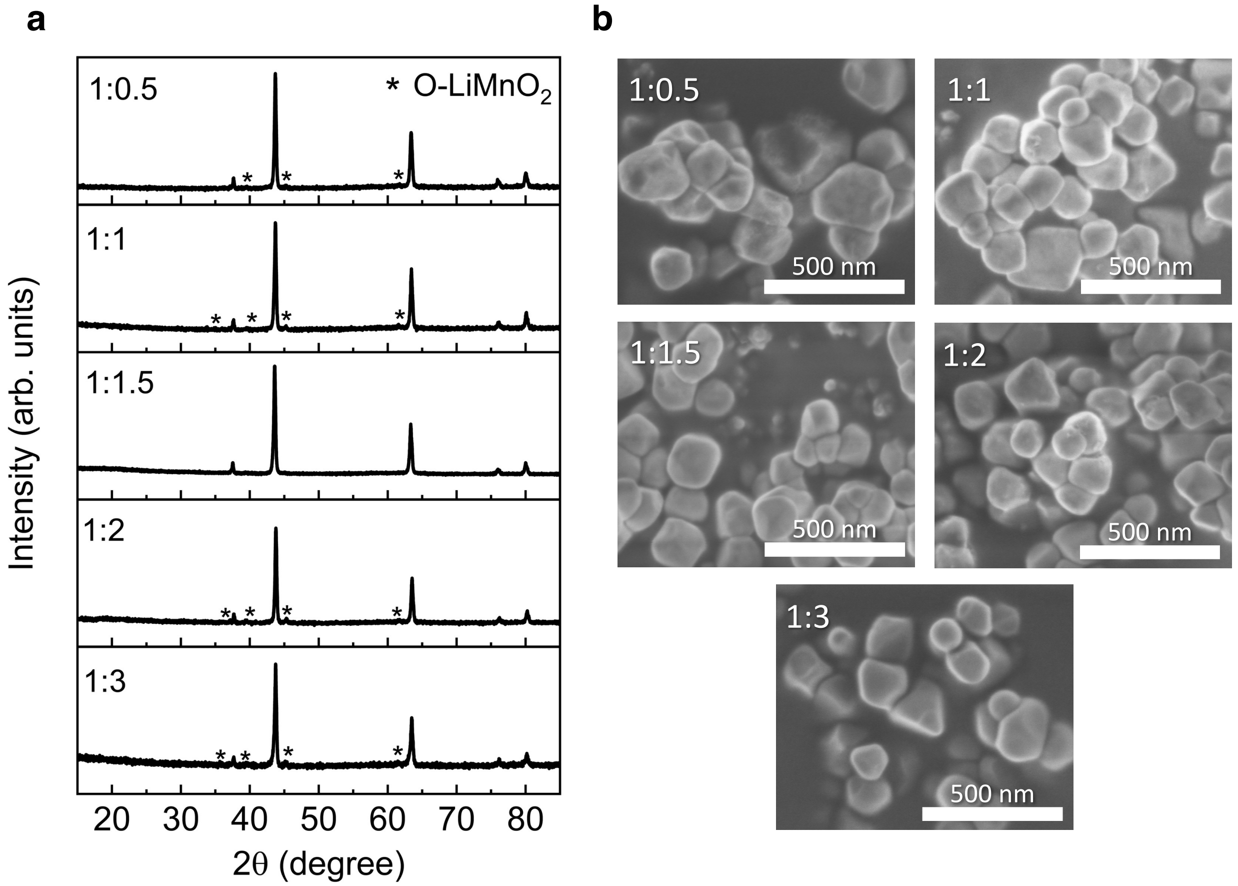

Supplementary Figure 2 | The effect of the precursor-to-salt weight ratio on the NM synthesis of LMTO (using CsBr as the salt, calcined at 800°C for 5 minutes and annealed at 600°C for 20 hours, followed by DIW washing and Li-reinsertion). a XRD patterns and **b** SEM images of NM-LMTO synthesized with different precursor-to-salt ratios (e.g., 1:0.5, 1:1, 1:1.5, 1:2, and 1:3). The 1:1.5 weight ratio yields the purest LMTO phase within the collection, without notable $LiMnO_2$ impurity. The SEM images indicate that a higher precursor-to-salt ratio generally reduces the primary particle size under the same NM heating protocol. As the 1:1.5 ratio produces the purest LMTO phase with an acceptably small particle size under the optimized NM heating protocol, this ratio was established as the baseline for studying other NM synthesis parameters in the main manuscript. Source data for Fig. S2a are provided as a Source Data file.

a

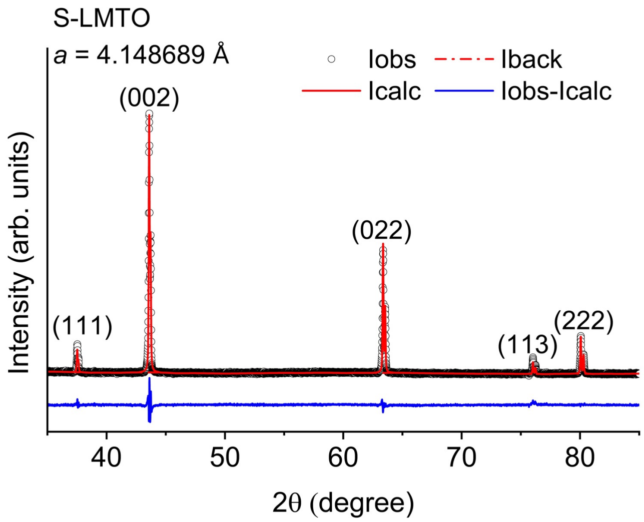

b

Space group: Fm-3m	(*u,v,w*) = (0.001086, 0.000157, 0.00655)					
**Atom**	**Wyckoff position**	***x***	***y***	***z***	**Occupancy**	B_{iso}
Li	4a	0	0	0	0.58043	1.57038
Mn	4a	0	0	0	0.20156	0.49997
Ti	4a	0	0	0	0.20099	0.49997
O1	4b	0.5	0.5	0.5	1	1.40575
a=*b*=*c*=4.148689Å Volume=71.4057Å^3 derived density=3.79 kg/l						
R_{wp}=6.07546, Goodness of fit=1.14855						

c

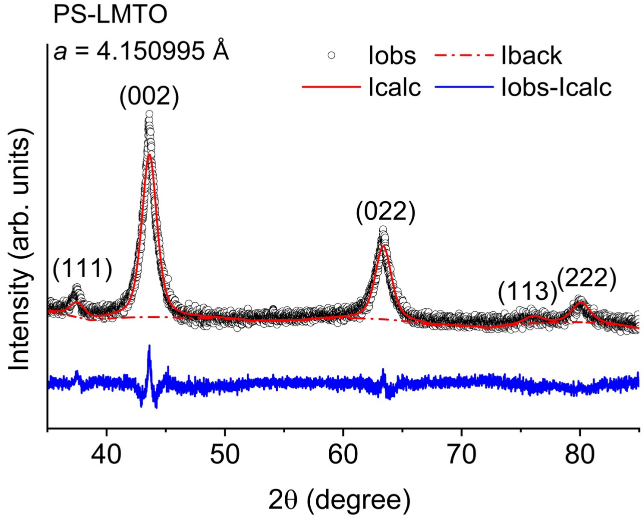

d

Space group: Fm-3m	(*u,v,w*) = (1.543494, 0.141838, 1.924958)					
**Atom**	**Wyckoff position**	***x***	***y***	***z***	**Occupancy**	B_{iso}
Li	4a	0	0	0	0.59704	2.45302
Mn	4a	0	0	0	0.20379	0.47062
Ti	4a	0	0	0	0.20658	0.47062
O1	4b	0.5	0.5	0.5	1	1.45982
a=*b*=*c*=4.150995Å Volume=71.5247Å^3 derived density=3.83 kg/l						
R_{wp}=5.61758, Goodness of fit=1.13044						

Supplementary Figure 3 | Structural comparison of LMTO synthesized by solid-state and pulverization methods. The XRD refinements and structural parameters of the (**a, b**) solid state synthesized LMTO (S-LMTO) and (**c, d**) pulverized LMTO (PS-LMTO). The crystallographic data for $LiFeO_2$ (ICSD collection code 51208) in the $Fm\bar{3}m$ space group used as a reference input file. A Pseudo-Voigt profile was employed. Initially, the lattice parameters were refined using the atomic proportions from the chemical composition of $Li_{1.2}Mn_{0.4}Ti_{0.4}O_2$ (LMTO) as the starting atomic occupancies. Subsequently, both the lattice parameters and atomic occupancies were refined in conjunction. The initial B_{iso} values were set at 2.5 for Li, 0.5 for transition metals, and 1.5 for oxygen. Then, we refined the B_{iso} values for the transition metals (e.g., Mn, Ti), ensuring they remained identical and varied together. Subsequently, we refined the B_{iso} values for Li and then for O. The same refinement procedure was applied to the XRD refinements presented in the Supplementary Information. Source data for Figs. S3a and S3c are provided as a Source Data file.

a

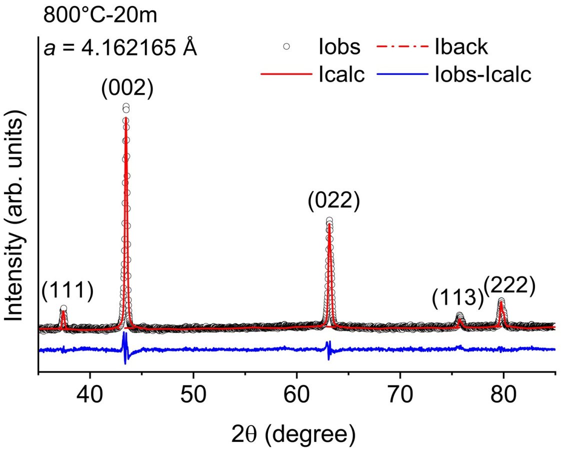

b

Space group: Fm-3m (*u,v,w*) = (0.010033, 0.001172, 0.038668)

Atom	Wyckoff position	*x*	*y*	*z*	Occupancy	B_{iso}
Li	4a	0	0	0	0.57142	1.64498
Mn	4a	0	0	0	0.19894	0.49726
Ti	4a	0	0	0	0.19893	0.49726
O1	4b	0.5	0.5	0.5	1	1.32699

a=*b*=*c*=4.162165Å Volume=72.1037Å^3 derived density=3.72 kg/l

R_{wp}=6.44342, Goodness of fit=1.22431

c

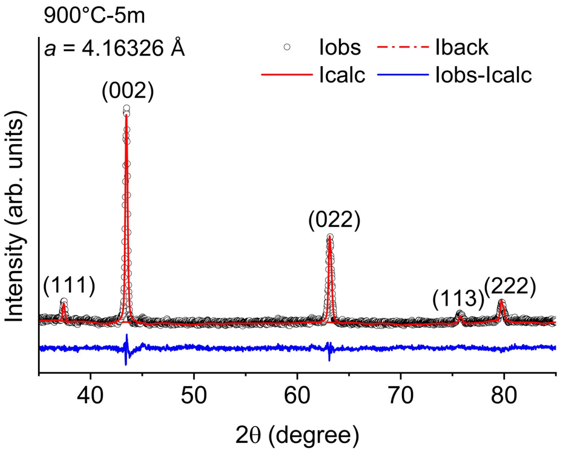

d

Space group: Fm-3m (*u,v,w*) = (0.044786, 0.006448, 0.0339)

Atom	Wyckoff position	*x*	*y*	*z*	Occupancy	B_{iso}
Li	4a	0	0	0	0.57535	1.69464
Mn	4a	0	0	0	0.20342	0.49817
Ti	4a	0	0	0	0.20241	0.49817
O1	4b	0.5	0.5	0.5	1	1.46554

a=*b*=*c*=4.16326Å Volume=72.1607Å^3 derived density=3.76 kg/l

R_{wp}=7.58757, Goodness of fit=1.16993

e

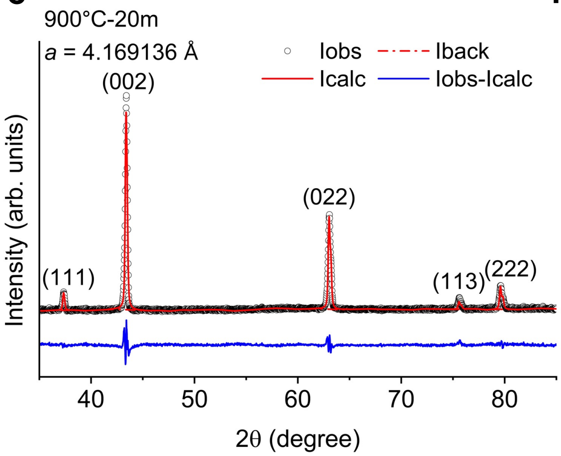

f

Space group: Fm-3m (*u,v,w*) = (0.019885, 0.000055, 0.02819)

Atom	Wyckoff position	*x*	*y*	*z*	Occupancy	B_{iso}
Li	4a	0	0	0	0.57110	1.59033
Mn	4a	0	0	0	0.19896	0.49739
Ti	4a	0	0	0	0.19912	0.49739
O1	4b	0.5	0.5	0.5	1	1.29941

a=*b*=*c*=4.169136Å Volume=72.4666Å^3 derived density=3.71 kg/l

R_{wp}=6.75792, Goodness of fit=1.27073

Supplementary Figure 4 | Effect of calcination temperature and time on the structure of NM-LMTO. The XRD refinements and structural parameters of the (**a, b**) NM-LMTO calcined at 800°C for 20 minutes, (**c, d**) NM-LMTO calcined at 900°C for 5 minutes, and (**e, f**) NM-LMTO calcined at 900°C for 20 minutes. All samples were subjected to argon annealing at 600°C for 20 hours after calcination. Source data for Figs. S4a, S4c, and S4e are provided as a Source Data file.

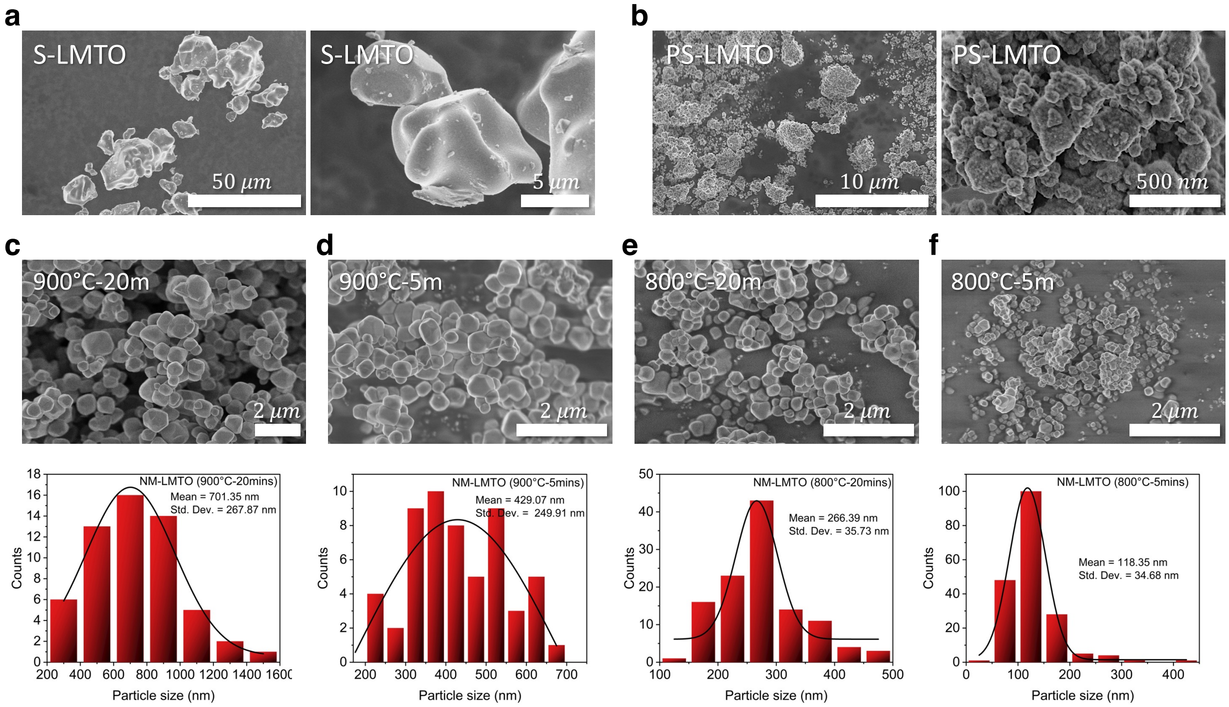

Supplementary Figure 5 | Comparison of particle morphology and size for LMTO synthesized via different methods. (**a, b**) SEM images of **a** S-LMTO and **b** PS-LMTO. (**c–f**) SEM images and particle size distribution plots of NM-LMTO calcined at **c** 900°C for 20 minutes, **d** 900°C for 5 minutes, **e** 800°C for 20 minutes, and **f** 800°C for 5 minutes: after the calcination, the NM-LMTO particles underwent annealing at 600°C for 20 hours in Argon. The precursor-to-salt (CsBr) weight ratio was 1:1.5. Source data for Figs. S5c–S5f are provided as a Source Data file.

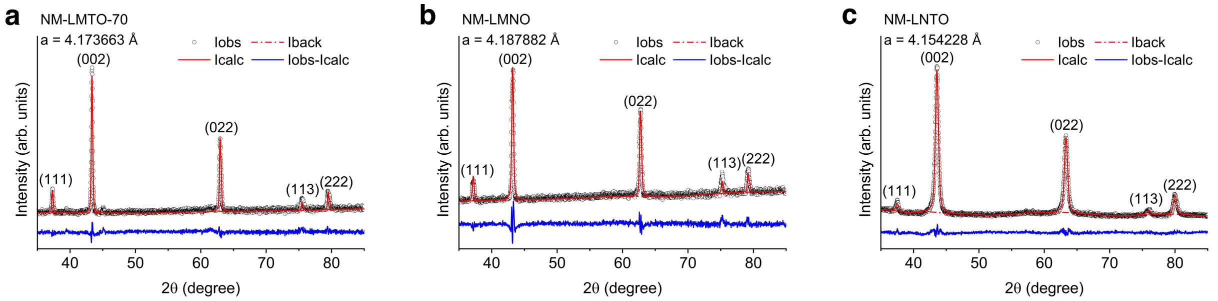

d

Space group: Fm-3m	(u,v,w) = (0.01096, 0.001714, 0.039476)					
Atom	Wyckoff position	x	y	z	Occupancy	B_{iso}
Li	4a	0	0	0	0.54510	0.89790
Mn	4a	0	0	0	0.35215	0.49727
Ti	4a	0	0	0	0.10275	0.49727
O	4b	0.5	0.5	0.5	1	1.20444
$a=b=c$=4.173663Å Volume=72.7029Å^3 derived density=4.02 kg/l						
R_{wp}=8.16834, Goodness of fit=1.11501						

e

Space group: Fm-3m	(u,v,w) = (-0.03068, --0.004487, 0.088836)					
Atom	Wyckoff position	x	y	z	Occupancy	B_{iso}
Li	4a	0	0	0	0.56274	0.89998
Mn	4a	0	0	0	0.29564	0.49123
Nb	4a	0	0	0	0.09375	0.49123
O	4b	0.5	0.5	0.5	1	1.36679
$a=b=c$=4.187882Å Volume=73.4485Å^3 derived density=4.06 kg/l						
R_{wp}=7.55477, Goodness of fit=1.07848						

f

Space group: Fm-3m	(u,v,w) = (-0.00231, 0.011185, 0.282522)					
Atom	Wyckoff position	x	y	z	Occupancy	B_{iso}
Li	4a	0	0	0	0.59513	2.54147
Ni	4a	0	0	0	0.09866	0.53431
Ti	4a	0	0	0	0.28801	0.53431
O	4b	0.5	0.5	0.5	1	0.93419
$a=b=c$=4.154228Å Volume=71.6920Å^3 derived density=3.68 kg/l						
R_{wp}=9.19755, Goodness of fit=1.04248						

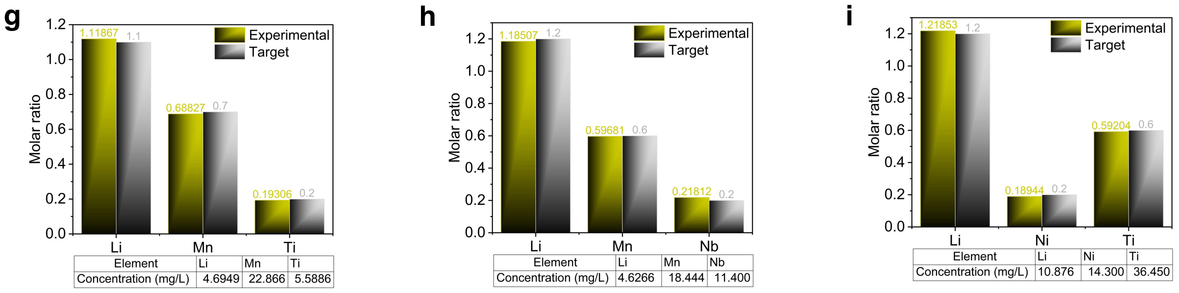

Element	Li	Mn	Ti
Concentration (mg/L)	4.6949	22.866	5.5886

Element	Li	Mn	Nb
Concentration (mg/L)	4.6266	18.444	11.400

Element	Li	Ni	Ti
Concentration (mg/L)	10.876	14.300	36.450

Supplementary Figure 6 | Phase and compositional analysis of NM-LMTO-70, NM-LMNO, and NM-LNTO synthesized by NM method. (**a–c**) The XRD patterns of **a** NM-$Li_{1.1}Mn_{0.7}Ti_{0.2}O_2$ (NM-LMTO-70: 900°C for 1 minute calcination & 600°C for 5 hours annealing in argon), **b** NM-$Li_{1.2}Mn_{0.6}Nb_{0.2}O_2$ (NM-LMNO: 950°C for 3 minutes calcination & 600°C for 5 hours annealing in argon), and **c** NM-$Li_{1.2}Ni_{0.2}Ti_{0.6}O_2$ (NM-LNTO, 750°C for 5 minutes calcination & 600°C for 5 hours annealing in air). These samples underwent Li-reinsertion after the DIW washing of CsBr. (**d–f**) The XRD refinement table of **d** NM-LMTO-70, **e** LMNO, and **f** LNTO. The ICP-OES results of **g** NM-LMTO-70, **h** LMNO, and **i** LNTO. Source data for Figs. S6a–S6c and S6g–S6i are provided as a Source Data file.

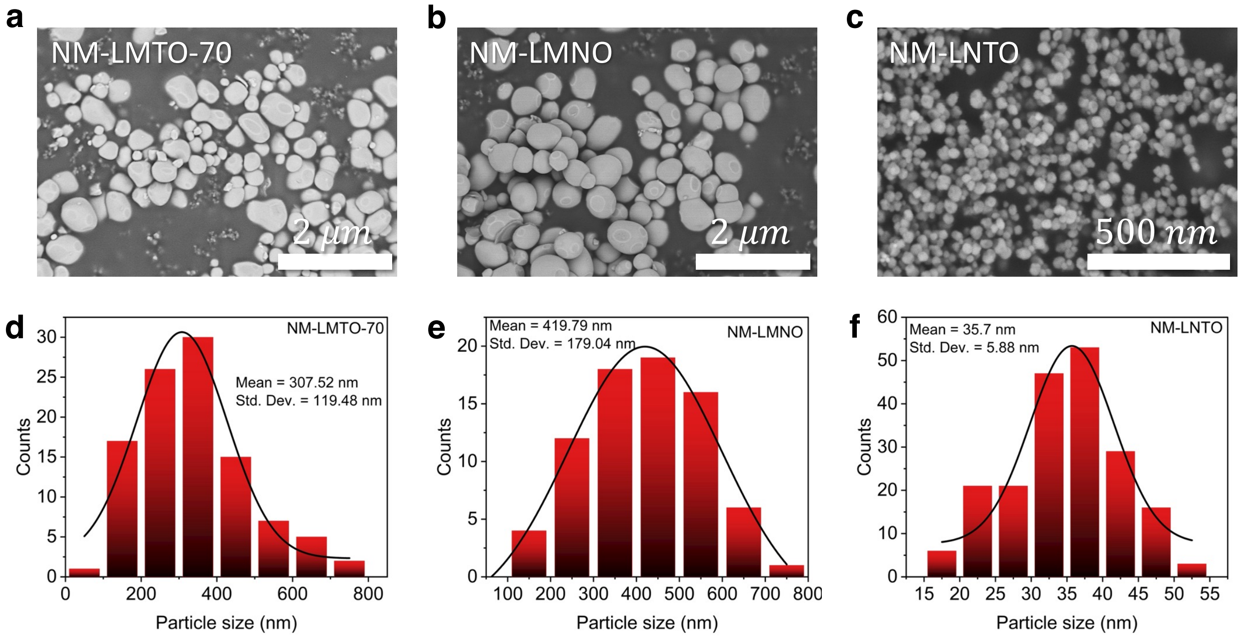

Supplementary Figure 7 | Morphology and particle size distribution of NM-derived LMTO-70, LMNO, and LNTO particles. SEM images of **a** NM-LMTO-70, **b** NM-LMNO, and **c** NM-LNTO particles. The particle size distribution of **d** NM-LMTO-70, **e** NM-LMNO, and **f** NM-LNTO particles. Source data for Figs. S7d–S7f are provided as a Source Data file.

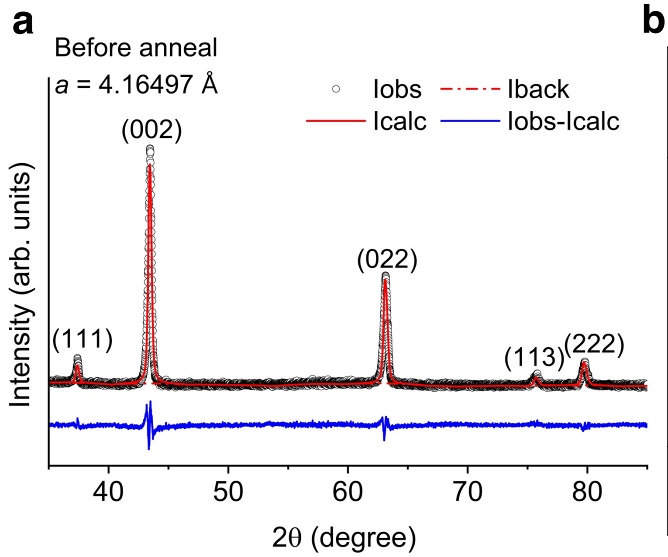

b

Space group: Fm-3m (*u*,*v*,*w*) = (0.037723, 0.003055, 0.078474)

Atom	Wyckoff position	*x*	*y*	*z*	Occupancy	B_{iso}
Li	4a	0	0	0	0.58398	2.04839
Mn	4a	0	0	0	0.20101	0.49911
Ti	4a	0	0	0	0.19717	0.49911
O	4b	0.5	0.5	0.5	1	1.37808

a=*b*=*c*=4.16497Å Volume=72.2467Å^3 derived density=3.73 kg/l

R_{wp}=6.25277, Goodness of fit=1.23702

Supplementary Figure 8 | XRD refinement of NM-LMTO without argon annealing. a The XRD refinement and **b** structural parameters of NM-LMTO calcined at 800°C for 5 minutes without argon annealing. Source data for Fig. S8a are provided as a Source Data file.

a

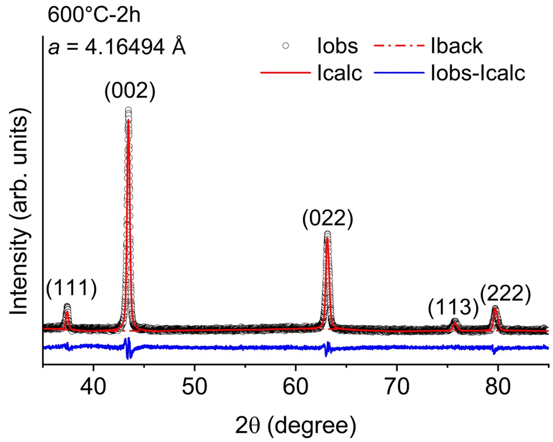

b

Space group: Fm-3m **(*u,v,w*) = (0.044022, 0.003473, 0.071273)**

Atom	Wyckoff position	*x*	*y*	*z*	Occupancy	B_{iso}
Li	4a	0	0	0	0.591	0.5
Mn	4a	0	0	0	0.206	0.5
Ti	4a	0	0	0	0.203	0.5
O	4b	0.5	0.5	0.5	1	0.8736

a=*b*=*c*=4.16494Å Volume=72.25Å^3 derived density=3.78 kg/l

R_{wp}=6.2687, Goodness of fit=1.29074

c

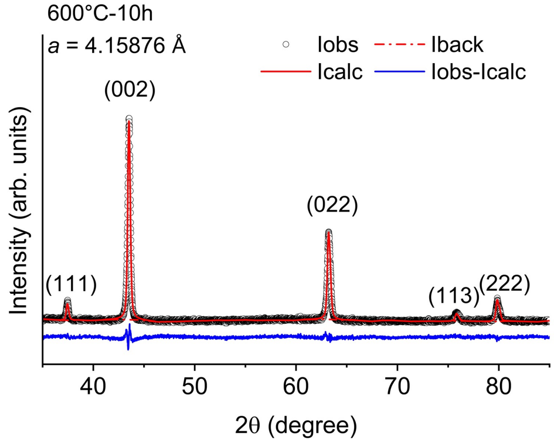

d

Space group: Fm-3m **(*u,v,w*) = (0.026009, 0.002469, 0.060762)**

Atom	Wyckoff position	*x*	*y*	*z*	Occupancy	B_{iso}
Li	4a	0	0	0	0.595	0.5
Mn	4a	0	0	0	0.202	0.5
Ti	4a	0	0	0	0.202	0.5
O	4b	0.5	0.5	0.5	1	0.7717

a=*b*=*c*=4.15876Å Volume=71.93Å^3 derived density=3.78 kg/l

R_{wp}=5.5877, Goodness of fit=1.11737

e

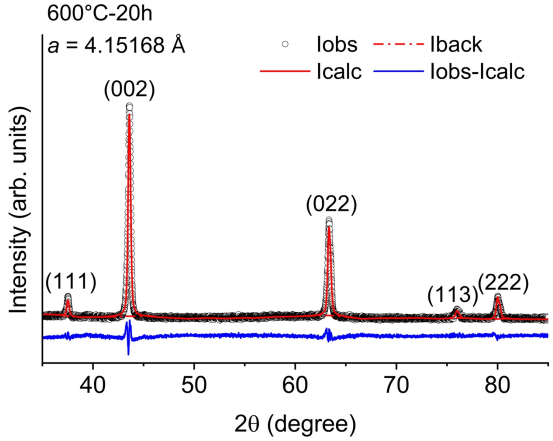

f

Space group: Fm-3m **(*u,v,w*) = (0.020021, 0.004059, 0.062948)**

Atom	Wyckoff position	*x*	*y*	*z*	Occupancy	B_{iso}
Li	4a	0	0	0	0.599	0.5
Mn	4a	0	0	0	0.199	0.5
Ti	4a	0	0	0	0.200	0.5
O	4b	0.5	0.5	0.5	1	0.5702

a=*b*=*c*=4.15168Å Volume=71.56Å^3 derived density=3.78 kg/l

R_{wp}=5.6633, Goodness of fit=1.16895

Supplementary Figure 9 | Effect of annealing duration on NM-LMTO crystal structure. The XRD refinements and structural parameters of (**a, b**) NM-LMTO annealed at 600°C for 2 hours, (**c, d**) NM-LMTO annealed at 600°C for 10 hours, and (**e, f**) NM-LMTO annealed at 600°C for 20 hours. All samples were calcined at 800°C for 5 minutes prior to annealing. Source data for Figs. S9a, S9c, and S9e are provided as a Source Data file.

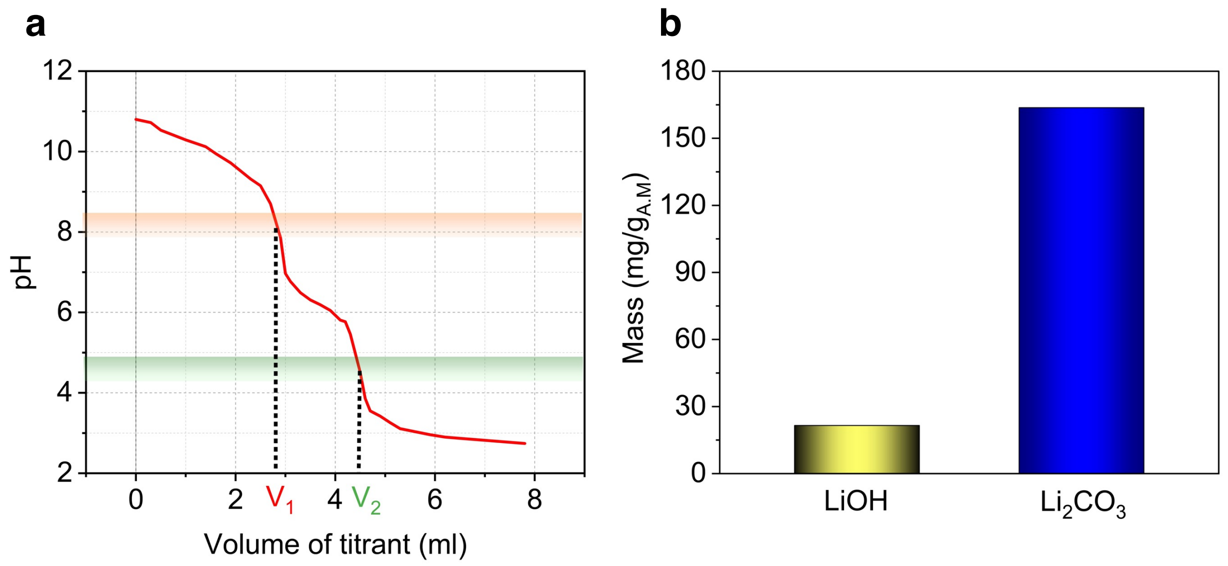

Supplementary Figure 10 | Detection of dissolved Li-species from LMTO due to Li^{+}/H^{+} exchange. a pH titration: A 10 mL sample of the washing solution (collected after washing the NM-LMTO powder following calcination in 1000 mL deionized water to remove CsBr) was titrated with 0.01 M HCl (titrant). The pH of the solution was continuously measured using a pH meter (Orion Star A211 Benchtop pH Meter equipped with a Thermo Scientific Orion 9107BN Triode 3-in-1 pH probe, featuring automatic temperature compensation) as HCl was added, allowing for the detection of endpoints corresponding to the neutralization of basic species (LiOH and Li_2CO_3). The volumes V_1 and V_2 corresponding to these endpoints were obtained from the titration curve. **b** Calculation of Li_2CO_3 and LiOH amounts: The amounts of Li_2CO_3 and LiOH were calculated based on the titration volumes V_1 and V_2. The volume equivalent to Li_2CO_3 is calculated as $2(V_2 – V_1)$, and the volume equivalent to LiOH is calculated as $2V_1 – V_2$. These values were then used to determine the mass of Li_2CO_3 and LiOH in the entire washing solution, and the results were normalized to 1.5 g, the weight of the NM-LMTO collected after vacuum filtration of the washing solution. Source data for Figs. S10a and S10b are provided as a Source Data file.

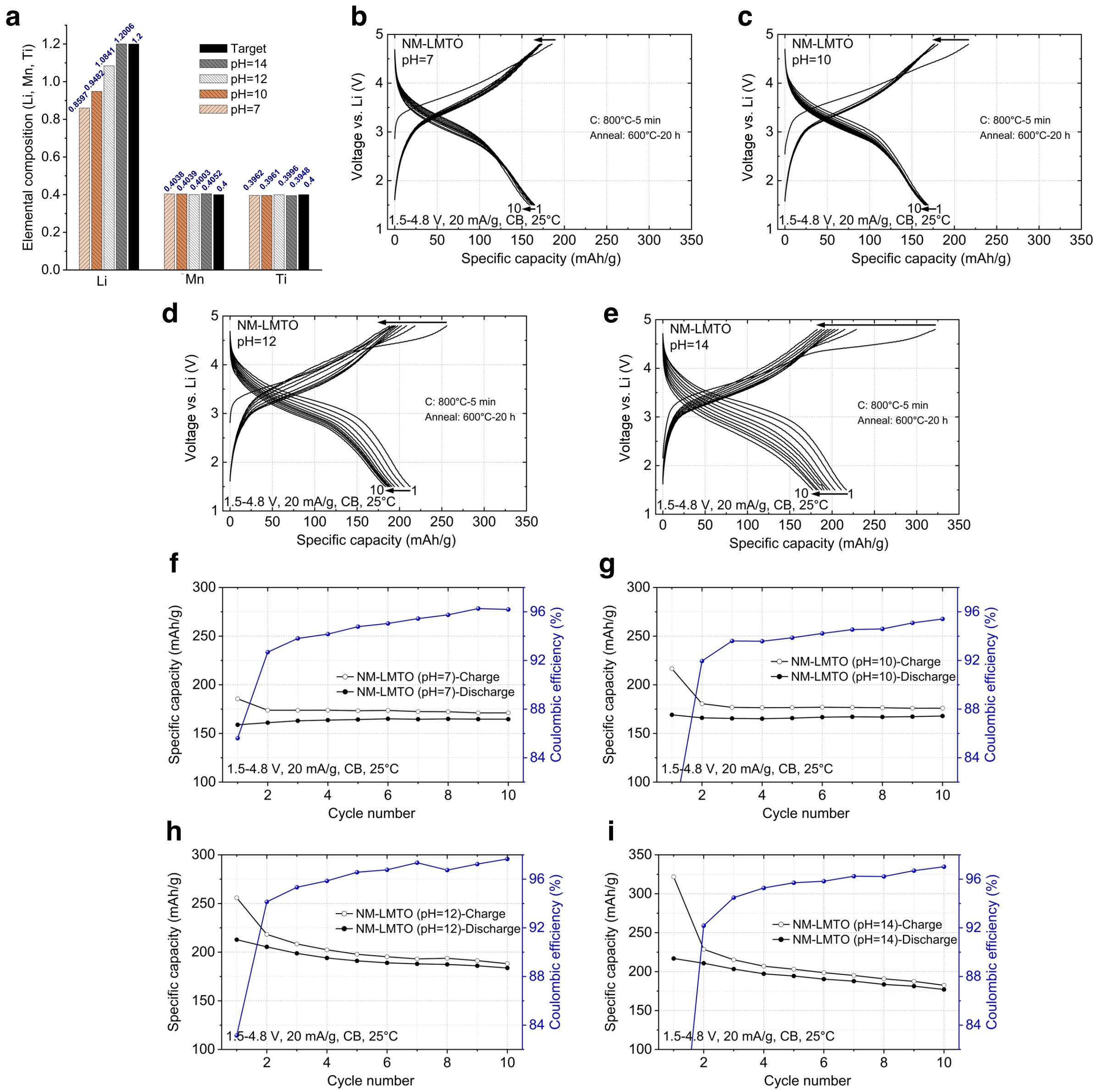

Supplementary Figure 11 | Effect of LiOH washing pH on lithium content and electrochemical performance. a Elemental analysis of NM-LMTO samples calcined at 800°C for 5 minutes and annealed at 600°C for 10 hours with the precursor-to-salt ratio of 1:1.5, then washed with LiOH solutions at varying pH levels, as determined by ICP-OES. The amount of Li detected from LMTO is significantly lower than expected when NM-LMTO is washed with pH 7 DIW. Increasing the pH of the washing solution with higher amounts of LiOH recovers the Li content in LMTO, indicating a mitigation of Li^+/H^+ exchange. (**b–e**) Voltage profiles of NM-LMTO samples in a Li||NM-LMTO cell (calcined at 800°C for 5 minutes and annealed at 600°C for 20 hours) washed with LiOH solutions of varying pH: **b** pH 7 (DI water), **c** pH 10, **d** pH 12, and **e** pH 14. Cycling was performed between 1.5–4.8 V at 20 mA/g in electrodes with a 70:20(CB):10 composition. (**f–i**) Ten-cycle capacity retention and coulombic efficiency for the corresponding samples. Source data for Figs. S11a–S11i are provided as a Source Data file.

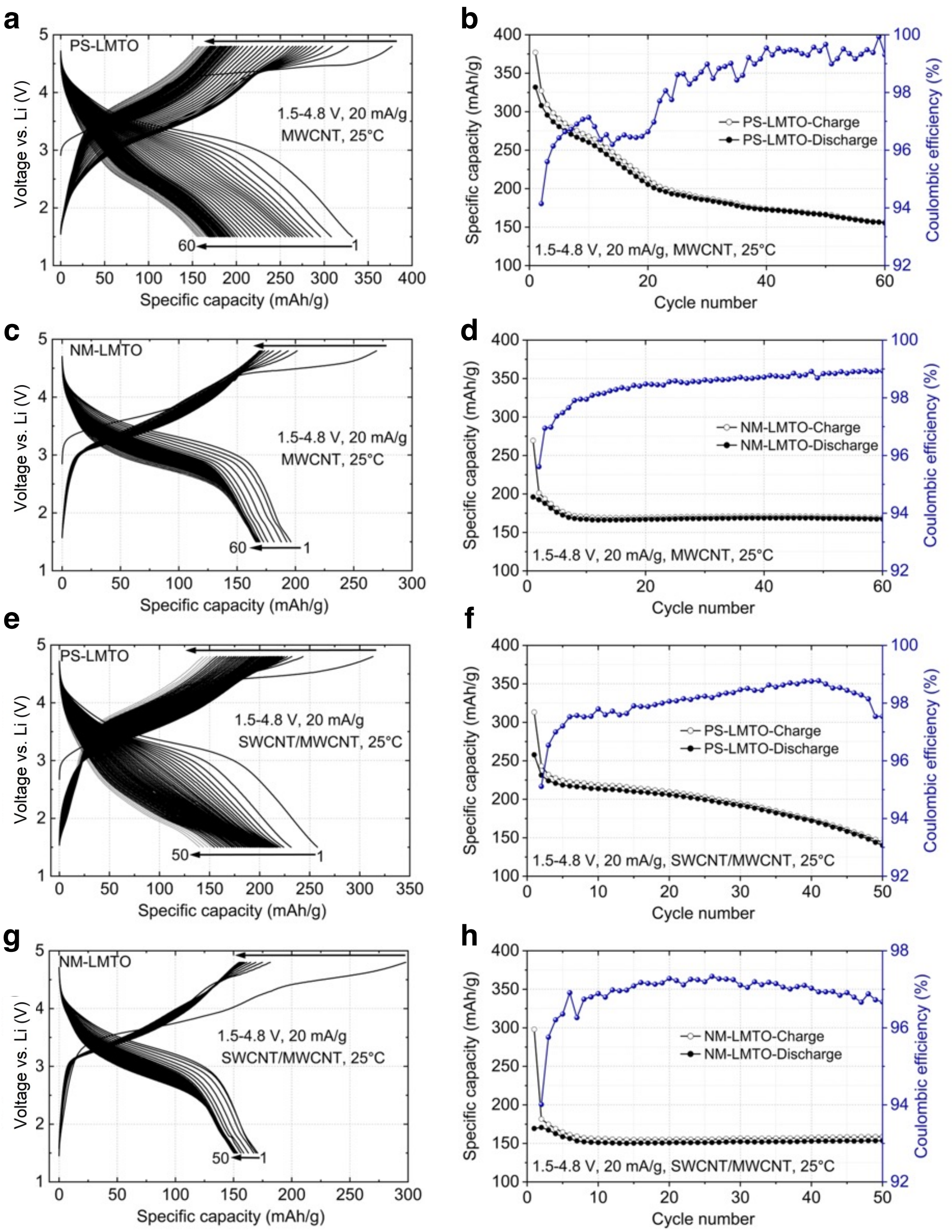

Supplementary Figure 12 | Electrochemical profiles of PS- and NM-LMTO with different carbon additives. **a** Voltage profiles, **b** capacity retention, and coulombic efficiency of PS-LMTO (90:5(MWCNT):5 electrode) when cycled in a Li||PS-LMTO cell between 1.5-4.8 V at 20 mA/g. **c** Voltage profiles, **d** capacity retention, and coulombic efficiency of NM-LMTO (90:5(MWCNT):5 electrode) when cycled in a Li||NM-LMTO cell between 1.5-4.8 V at 20 mA/g. **e** Voltage profiles, **f** capacity retention, and coulombic efficiency of PS-LMTO in a direct slurry mixed 85:2.5(SWCNT):7.5(MWCNT):5 electrode in a Li||PS-LMTO cell. **g** Voltage profiles, **h** capacity retention, and coulombic efficiency of NM-LMTO in a direct slurry mixed 85:2.5(SWCNT):7.5(MWCNT):5 electrode in a Li||NM-LMTO cell. Source data for Figs. S12a–S12h are provided as a Source Data file.

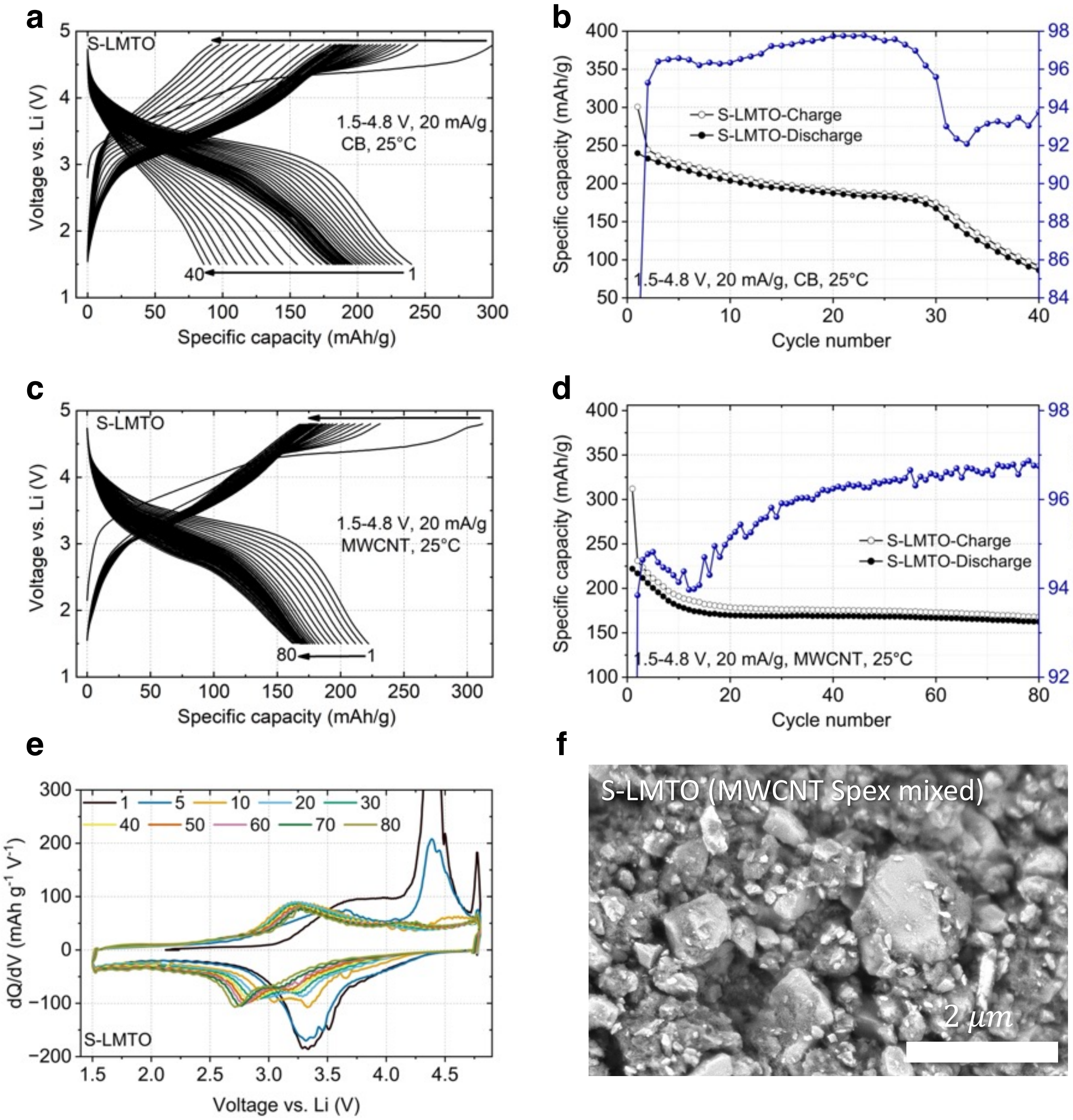

Supplementary Figure 13 | Impact of mixing and carbon type on S-LMTO electrochemical performance. **a** Voltage profile, **b** capacity retention, and coulombic efficiency of S-LMTO electrodes (70:20(CB):10) in a Li||S-LMTO cell, with S-LMTO and CB mixed for 2 hours using a Spex mill. **c** Voltage profile, **d** capacity retention, and coulombic efficiency of S-LMTO electrodes (70:20(MWCNT):10) in a Li||S-LMTO cell, with S-LMTO and MWCNT mixed for 1 hour using a Spex mill. Both cells were cycled between 1.5 and 4.8 V at 20 mA/g. **e** The dQ/dV plots over various cycles of S-LMTO-MWCNT-PVDF electrode in a Li||S-LMTO cell in Figure C, with legends indicating the corresponding cycle numbers. **f** The SEM image of the S-LMTO/MWCNT mixture**.** Source data for Figs. S13a–S13e are provided as a Source Data file.

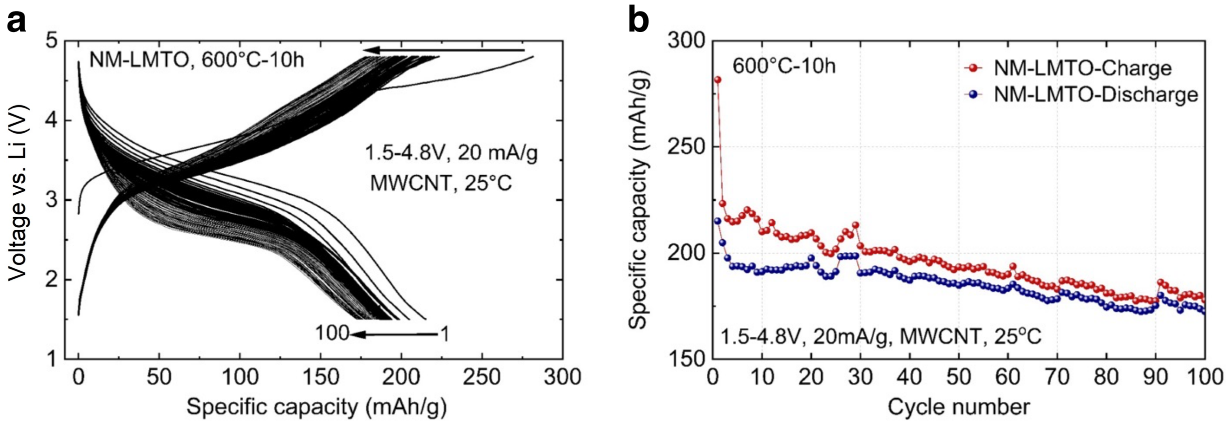

Supplementary Figure 14 | The performance of NM-LMTO in the 70-LMTO:20-MWCNT:10-PVDF electrode. a Voltage profile and **b** capacity retention of NM-LMTO calcined at 800°C for 5 minutes and annealed at 600°C for 10 hours, followed by DIW washing and Li-reinsertion. The material was tested at 20 mA/g over a voltage range of 1.5–4.8 V in a Li||NM-LMTO cell. Source data for Figs. S14a and S14b are provided as a Source Data file.

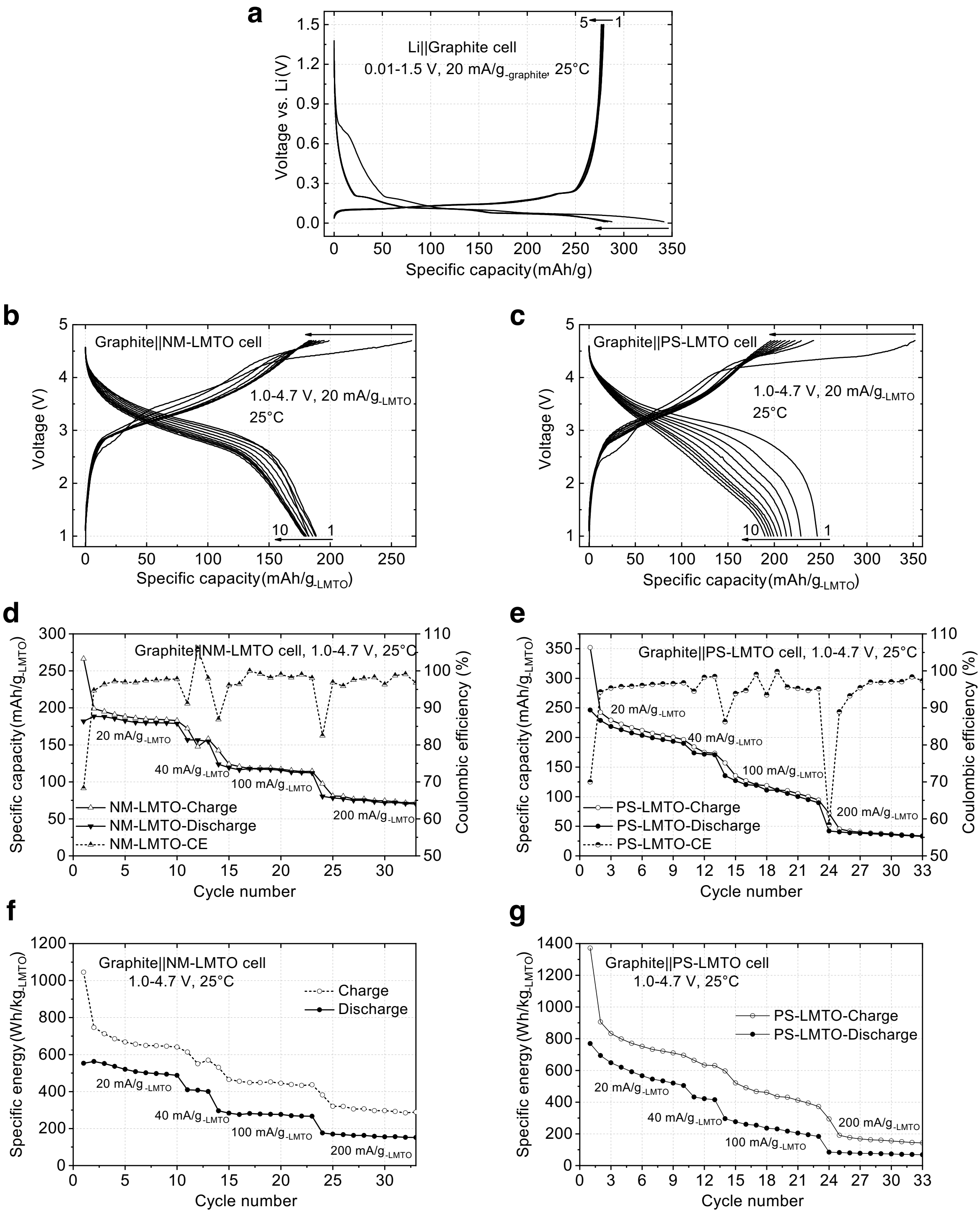

Supplementary Figure 15 | Electrochemical performance of Graphite||NM/PS-LMTO full cells. a Initial 5-cycle voltage profile of the graphite electrode composed of 93.2 wt% graphite (AA40798TC, Alfa Aesar), 2.5 wt% carbon black, and 4.3 wt% PVDF, cycled in a Li||Graphite cell between 0.01–1.5 V at 20 mA/g at 25°C. (**b, c**) Initial 10-cycle voltage profiles of Graphite||NM/PS-LMTO cells: **b** Graphite||NM-LMTO(90:5-MWCNT:5) and **c** Graphite||PS-LMTO(90:5-MWCNT:5), cycled at 20 mA/g$_{-LMTO}$ between 1.0–4.7 V at 25°C.

(**d, e**) Specific capacity (mAh/g_{-LMTO}) retention and coulombic efficiency of **d** NM-LMTO and **e** PS-LMTO in the Graphite||NM/PS-LMTO cells, cycled at 20 mA/g_{-LMTO} for the initial 10 cycles, 40 mA/g_{-LMTO} for the next 3 cycles, 100 mA/g_{-LMTO} for the next 10 cycles, and 200 mA/g_{-LMTO} for the final 10 cycles, within a voltage window of 1.0–4.7 V at 25°C. (**f, g**) Corresponding specific energy (Wh/kg_{-LMTO}) retention of **f** NM-LMTO and **g** PS-LMTO in the Graphite||NM/PS-LMTO cells. Source data for Figs. S15a–S15g are provided as a Source Data file.

Supplementary Note 1 | Graphite||NM/PS-LMTO cell performance

We fabricated Graphite||NM-LMTO and Graphite||PS-LMTO coin cells using a 90(NM/PS-LMTO):5(MWCNT):5(PVDF) cathode and a 93.2(Graphite):2.5(CB):4.3(PVDF) anode. Supplementary Figure 15a presents the representative initial 5-cycle voltage profile of the graphite anode (AA40798TC, Alfa Aesar) with the 93.2:2.5:4.3 composition, cycled in a Li||Graphite cell between 0.01–1.5 V at 20 mA/g at room temperature. The graphite's initial discharge capacity reaches ~347 mA/g and the subsequent charging capacity to be ~280 mAh/g, showing slightly inferior capacities to what would be expected from an optimized graphite material (theoretical capacity of 372 mAh/g).

For Graphite||NM/PS-LMTO cell construction, the anode (N) and cathode (P) capacities were balanced to achieve an N/P ratio of approximately 1.2. This was based on the first discharge specific capacity of ~350 mAh/g for graphite (Supplementary Fig. 15a), the first charge capacity of ~270 mAh/g for NM-LMTO (Supplementary Fig. 12c), and ~380 mAh/g for PS-LMTO (Supplementary Fig. 12a), each measured at a current density of 20 mA/g in half-cell configurations. The areal loading of the PS/NM-LMTO was approximately 4 mg_{-LMTO}/cm^2, and the graphite anode loading was accordingly adjusted to maintain N/P ≈ 1.2 in the Graphite||NM/PS-LMTO cells.

Supplementary Figures 15b and 15c present the initial 10-cycle voltage profiles of the NM-LMTO and PS-LMTO in the Graphite||NM/PS-LMTO cells, respectively, cycled at 20 mA/g-LMTO between 1.0–4.7 V. In the full-cell configuration, the first discharge capacity of NM-LMTO in the 90:5:5 electrode is approximately 182 mAh/g_{-LMTO} (Supplementary Fig. 15b), which is slightly lower but comparable to the initial discharge capacity (~195 mAh/g_{-LMTO}) observed in the Li||NM-LMTO setup using the same electrode composition (Supplementary Fig. 12c). We note that the specific capacity of a cathode material in a full-cell is influenced not only by the typical factors such as cycling rate and voltage window (parameters that also apply to half-cells) but also by the anode material and the N/P ratio, both of which can affect the overall voltage profile and accessible capacity in a full-cell. Accordingly, NM-LMTO's capacity in the full-cell configuration may be further improved through optimization of these full-cell parameters.

The PS-LMTO electrode shows higher initial capacities than NM-LMTO in the full-cell (Supplementary Fig. 15c) as in the half-cell, yet its initial discharge capacity (~247 mAh/g_{-LMTO}) is quite lower than the value obtained in the half-cell (~320 mAh/g_{-LMTO}). This discrepancy can be attributed in part to lithium loss during the first cycle due to

irreversible lithium consumption at the graphite anode side. As shown in Supplementary Fig. 15a, our graphite anode exhibits significant initial lithium trapping upon lithiation (discharge in a Li||Graphite cell, charge in a Li||PS/NM-LMTO cell), likely due to SEI formation and other side reactions. This initial lithium loss reduces the lithium inventory available for reinsertion into the PS-LMTO during the first discharge in the Graphite||PS-LMTO cell, thereby limiting its capacity. In contrast, NM-LMTO already exhibits low initial coulombic efficiency in the Li||NM-LMTO cell (i.e., a large difference between charge and discharge capacities), suggesting that its capacity in a Graphite||NM-LMTO cell is less sensitive to lithium loss at the graphite side during the initial cycles.

The capacity retention of NM-LMTO (~94.8%) over the first 10 cycles at 20 mA/g_{-LMTO} in a Graphite||NM-LMTO cell is noticeably higher than that of PS-LMTO (~77.1%) (Supplementary Figs. 15b–e) in a Graphite||PS-LMTO cell, consistent with trends observed in Li||PS/NM-LMTO cell testing. As the cycling rate increases from 20 to 40, 100, and 200 mA/g_{-LMTO}, the discharge capacity of NM-LMTO gradually decreases to approximately 156, 120, and 77 mAh/g_{-LMTO}, respectively (Supplementary Fig. 15d). In contrast, the capacity of PS-LMTO drops to approximately 171, 110, and 38 mAh/g_{-LMTO} under the same conditions (Supplementary Fig. 15e), but its capacity retention is inferior to that of NM-LMTO in all rates. The corresponding specific energies (Wh/kg_{-LMTO}) for NM-LMTO and PS-LMTO obtained from the Graphite||NM/PS-LMTO cell tests are presented in Supplementary Figures 15f and 15g. Despite having a lower initial specific energy, NM-LMTO surpasses PS-LMTO in later cycles due to its enhanced cycling stability.

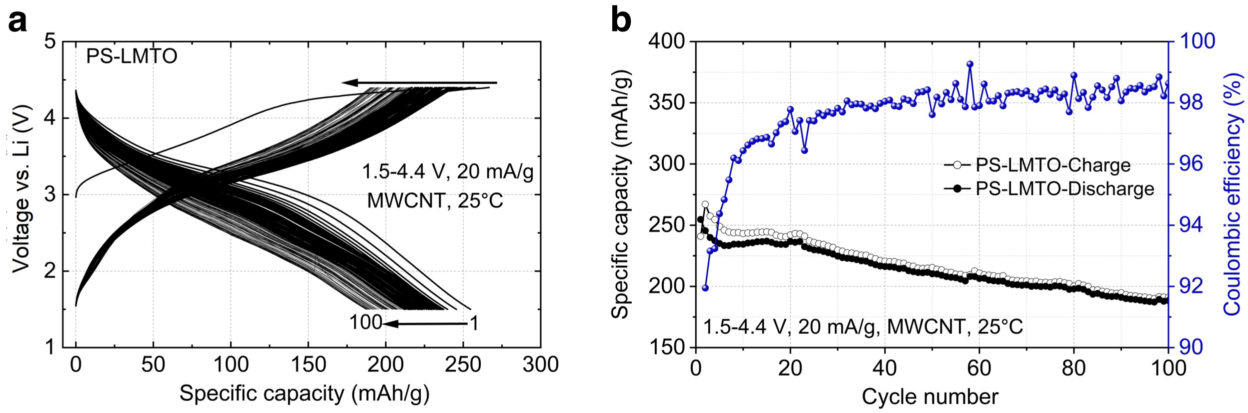

Supplementary Figure 16 | Voltage profile and cycling stability of PS-LMTO in a narrower voltage window. **a** Voltage profile, **b** capacity retention, and coulombic efficiency of PS-LMTO cycled in a Li||PS-LMTO cell between 1.5–4.4 V at 20 mA/g in a 70:20(MWCNT):10 composite electrode. Source data for Figs. S16a and S16b are provided as a Source Data file.

a

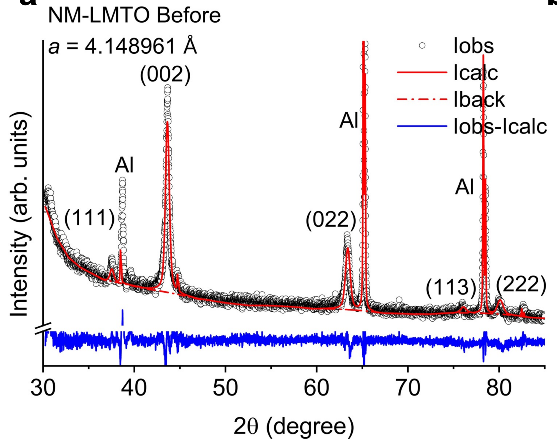

b

Space group: Fm-3m		(u,v,w) = (0.214723, 0.021668, 0.145189)				
Atom	Wyckoff position	x	y	z	Occupancy	B_{iso}
Li	4a	0	0	0	0.99235	2.35737
Mn	4a	0	0	0	0.19924	0.50018
Ti	4a	0	0	0	0.19895	0.50018
O1	4b	0.5	0.5	0.5	1	1.33109
$a=b=c$=4.148961Å Volume=71.4197Å^3 derived density=4.03 kg/l						
R_{wp}=10.03521, Goodness of fit=1.49886						

c

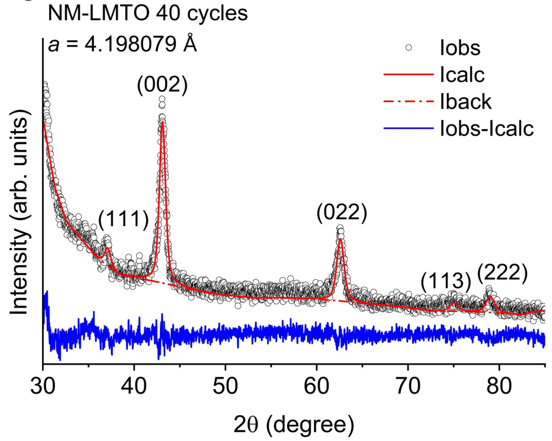

d

Space group: Fm-3m		(u,v,w) = (0.745383, 0.066257, 0.407668)				
Atom	Wyckoff position	x	y	z	Occupancy	B_{iso}
Li	4a	0	0	0	0.59768	2.64190
Mn	4a	0	0	0	0.19996	0.51349
Ti	4a	0	0	0	0.20073	0.51349
O	4b	0.5	0.5	0.5	1	1.72122
$a=b=c$=4.198079Å Volume=73.9864Å^3 derived density=3.66 kg/l						
R_{wp}=8.97358, Goodness of fit=0.96389						

e

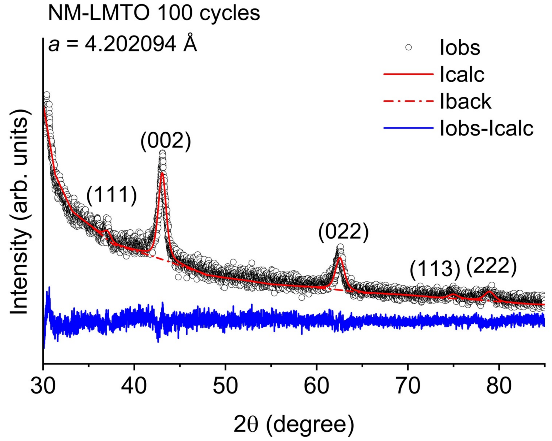

f

Space group: Fm-3m		(u,v,w) = (0.833418, 0.190158, 0.651816)				
Atom	Wyckoff position	x	y	z	Occupancy	B_{iso}
Li	4a	0	0	0	0.59901	2.43073
Mn	4a	0	0	0	0.20076	0.53517
Ti	4a	0	0	0	0.20039	0.53517
O	4b	0.5	0.5	0.5	0.98819	1.49975
$a=b=c$=4.202094Å Volume=74.1989Å^3 derived density=3.63 kg/l						
R_{wp}=6.96956, Goodness of fit=0.94907						

Supplementary Figure 17 | XRD refinement and structural parameters of NM-LMTO electrode films. (**a, b**) before cycling, (**c, d**) after 40 cycles, and (**e, f**) after 100 cycles in a Li||NM-LMTO cell. All samples were calcined at 800°C for 5 minutes and annealed at 600°C for 20 hours, followed by DIW washing and Li-reinsertion. The electrodes were prepared with a composition of 70:20:10 wt% (LMTO:MWCNT:PVDF) and cycled at 20 mA/g within a voltage window of 1.5–4.8 V. Source data for Figs. S17a, S17c, and S17e are provided as a Source Data file.

a

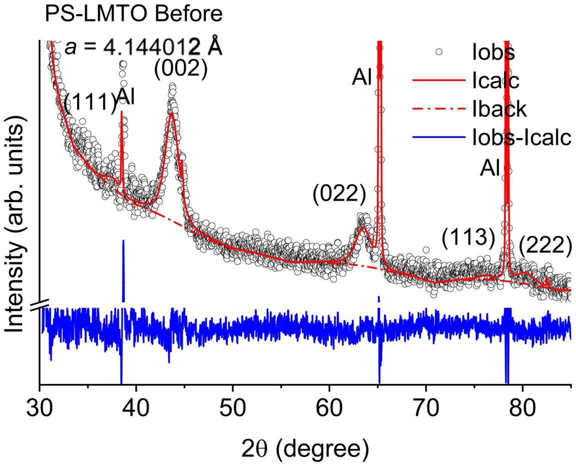

b

Space group: Fm-3m	(*u,v,w*) = (2.433329, 0.301095, 1.634513)					
**Atom**	**Wyckoff position**	***x***	***y***	***z***	**Occupancy**	B_{iso}
Li	4a	0	0	0	0.6	2.14762
Mn	4a	0	0	0	0.20413	0.49998
Ti	4a	0	0	0	0.20144	0.49998
O	4b	0.5	0.5	0.5	0.99142	1.35446
a=*b*=*c*=4.144012Å Volume=71.1638Å^3 derived density=3.82 kg/l						
R_{wp}=8.7648, Goodness of fit=1.105						

c

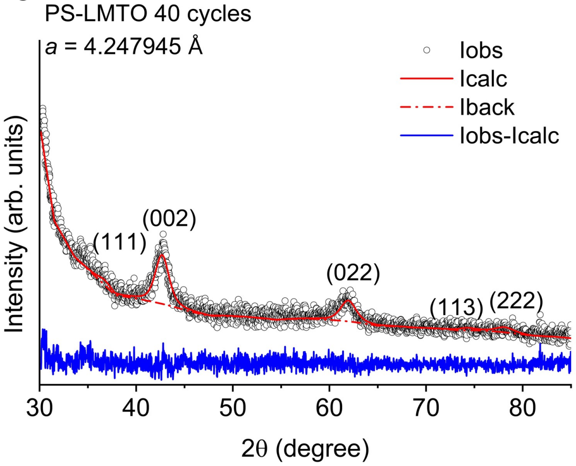

d

Space group: Fm-3m	(*u,v,w*) = (4.051905, 0.661027, 1.766866)					
**Atom**	**Wyckoff position**	***x***	***y***	***z***	**Occupancy**	B_{iso}
Li	4a	0	0	0	0.54256	2.10046
Mn	4a	0	0	0	0.20371	0.40445
Ti	4a	0	0	0	0.20544	0.40445
O	4b	0.5	0.5	0.5	0.97196	2.17562
a=*b*=*c*=4.247945Å Volume=76.65432Å^3 derived density=3.5 kg/l						
R_{wp}=9.18742, Goodness of fit=0.91704						

e

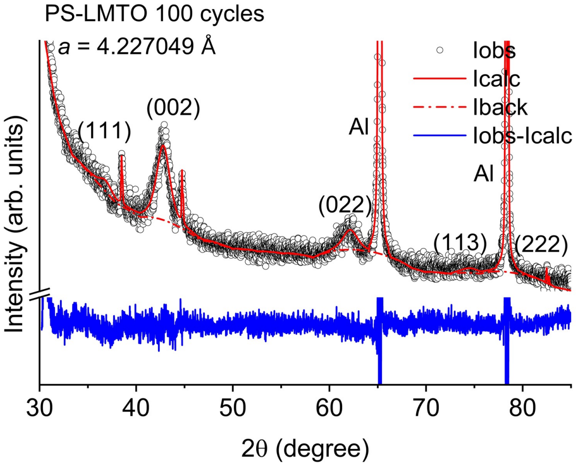

f

Space group: Fm-3m	(*u,v,w*) = (1.924619, 0.10408, 0.900788)					
**Atom**	**Wyckoff position**	***x***	***y***	***z***	**Occupancy**	B_{iso}
Li	4a	0	0	0	0.59978	6.43901
Mn	4a	0	0	0	0.20127	0.50561
Ti	4a	0	0	0	0.2011	0.50561
O	4b	0.5	0.5	0.5	0.94868	3.90278
a=*b*=*c*=4.227049Å Volume=75.52867Å^3 derived density=3.5 kg/l						
R_{wp}=7.87813, Goodness of fit=1.12685						

Supplementary Figure 18 | XRD refinements and structural parameters of PS-LMTO electrode films. (**a, b**) before cycling, (**c, d**) after 40 cycles, and (**e, f**) after 100 cycles in a Li||PS-LMTO cell. The electrodes were prepared with a composition of 70:20:10 wt% (LMTO:MWCNT:PVDF) and cycled at 20 mA/g within a voltage window of 1.5–4.8 V. Source data for Figs. S18a, S18c, and S18e are provided as a Source Data file.

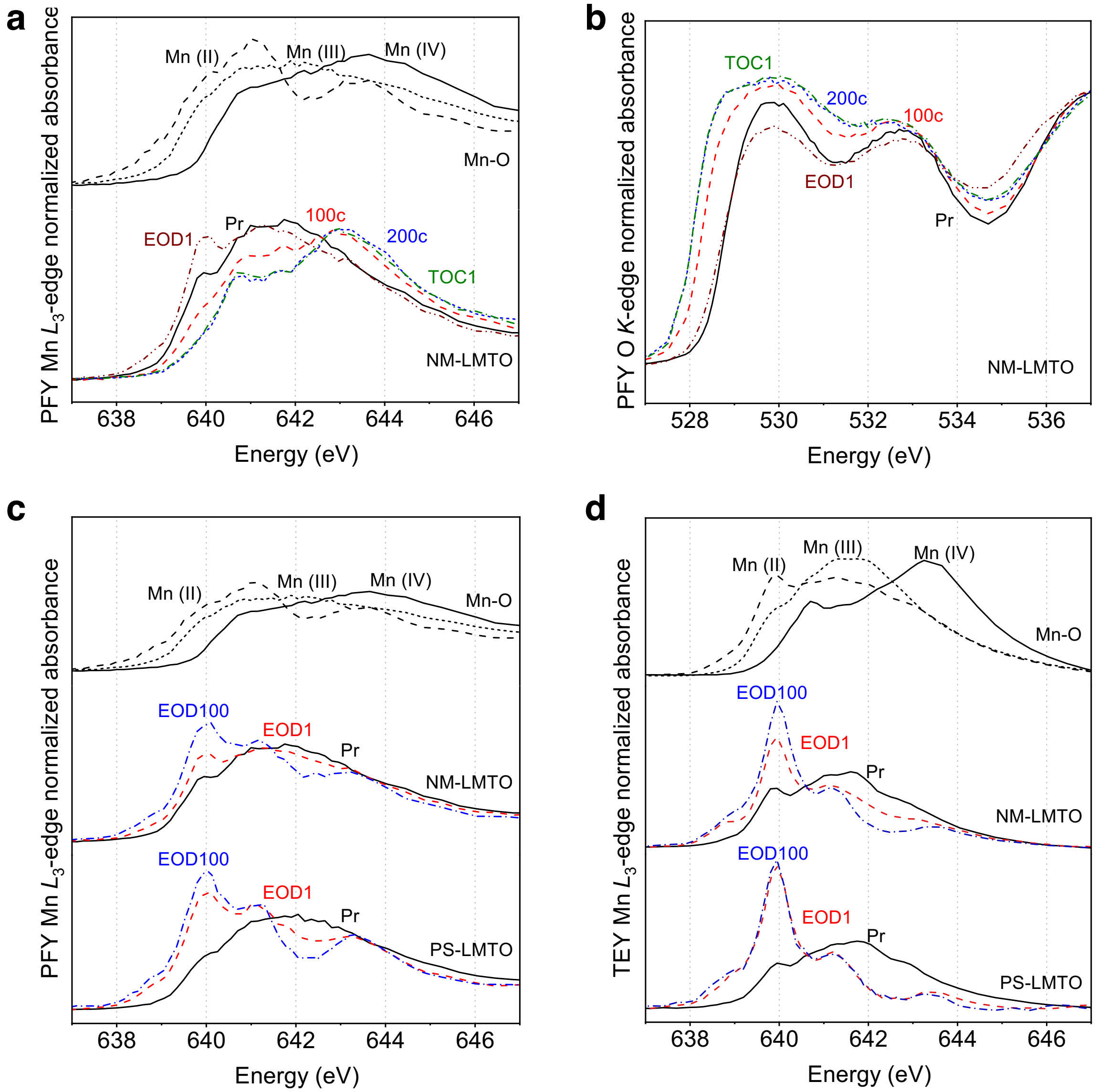

Supplementary Figure 19 | XAS analysis of Mn and O redox in NM/PS-LMTO during cycling. a Mn L_3-edge XAS spectra of the NM-LMTO electrode collected in Partial Fluorescence Yield (PFY) mode: before cycling (Pr), after the 1st charge to 100 mAh/g (100C), 200 mAh/g (200C), and 4.8 V (TOC1), and after the 1st discharge to 1.5 V (EOD1) at 20 mA/g in a Li||NM-LMTO cell. For reference, Mn L_3-edge spectra of MnO [Mn(II)], Mn_2O_3 [Mn(III)], and MnO_2 [Mn(IV)] are also included. **b** O *K*-edge XAS spectra of the NM-LMTO electrode collected in Partial Fluorescence Yield (PFY) mode: before cycling (Pr), after the 1st charge to 100 mAh/g (100C), 200 mAh/g (200C), and 4.8 V (TOC1), and after the 1st discharge to 1.5 V (EOD1) at 20 mA/g. (**c, d**) Mn L_3-edge XAS spectra of the NM-LMTO and PS-LMTO electrodes, collected in **c** Partial Fluorescence Yield (PFY) mode and **d** Total Electron Yield (TEY) mode, are shown: before cycling (Pr), after the 1st discharge (EOD1), and after the 100th discharge (EOD100) during 1.5–4.8 V cycling at 20 mA/g using Li||NM/PS-LMTO cells. Source data for Figs. S19a–S19d are provided as a Source Data file.

Supplementary Note 2 | Redox mechanism and oxygen loss comparison

Supplementary Fig. 19a shows the Mn L_3-edge XAS spectra of the NM-LMTO electrode collected in bulk-sensitive Partial Fluorescence Yield (PFY) mode: before cycling (Pr), after the 1st charge to 100 mAh/g (100C), 200 mAh/g (200C), and 4.8 V (TOC1), and after the 1st discharge to 1.5 V (EOD1) at 20 mA/g in a Li||NM-LMTO cell. For reference, Mn L_3-edge spectra of MnO [Mn(II)], Mn_2O_3 [Mn(III)], and MnO_2 [Mn(IV)] are also included. The Mn L_3-edge spectrum of NM-LMTO before cycling (Pr) closely resembles that of Mn_2O_3 (MnIII), indicating an initial Mn oxidation state near Mn^{3+}, consistent with its composition ($Li^{+}_{1.2}Mn^{3+}_{0.4}Ti^{4+}_{0.4}O^{2-}_{2}$). Upon the 1st charge to 100 mAh/g (100C) and 200 mAh/g (200C), the center of mass of the Mn L_3-edge shifts to higher energy, signifying Mn oxidation toward Mn^{4+}. Further charging to 4.8 V (TOC1) results in minimal change to the Mn edge, suggesting that additional capacity beyond this point involves negligible Mn oxidation.

It is widely understood that along with Mn oxidation, O oxidation takes place in LMTO (and other Li-excess Mn-DRX compounds) during charging. Supplementary Fig. 19b presents the pre-edge feature of the O *K*-edge XAS spectra of NM-LMTO collected in PFY mode, confirming O oxidation during charging. Upon the 1st charge to 100 mAh/g (100C), 200 mAh/g (200C), and 4.8 V (TOC1), an increasing intensity is observed between 527–529 eV, associated with Mn oxidation to Mn^{4+}. Concurrently, an intensity increase between 530–532 eV is detected, which is often attributed to O oxidation[1,2]. After the 1st discharge to 1.5 V, the pre-edge feature of the O *K*-edge spectra returns to a state similar to that before cycling.

This overall evolution in the Mn L_3-edge and O *K*-edge spectra demonstrates the oxidation of Mn and O during charging for NM-LMTO, which reverses upon discharging. This behavior is consistent with previous reports on LMTO in the literature. Due to limited time at the XAS facility, we did not conduct a separate analysis of Mn and O oxidation changes for PS-LMTO during the first cycle, as it was synthesized using the traditional method reported in the literature. However, numerous studies support a similar redox mechanism for such systems.

In parallel, we compared the Mn electronic states of NM-LMTO and PS-LMTO at different stages using both bulk-sensitive Partial Fluorescence Yield (PFY) mode and surface-sensitive Total Electron Yield (TEY) mode: before cycling (Pr), after the 1st discharge (EOD1), and after the 100th discharge (EOD100) (Supplementary Fig. 19c and 19d). The Mn L_3-edge spectra from both modes reveal a shift in the center of mass of the Mn edge toward lower energy after the first cycle, with a further shift after 100 cycles. This indicates a progressive decrease in the Mn oxidation state in the discharged state, from Mn^{3+} toward Mn^{2+} during cycling. However, this shift progresses more slowly in NM-LMTO for both modes. Notably, the PFY mode shows a stronger intensity between 641–643 eV after cycling, suggesting a higher retention of Mn^{3+} in the bulk of NM-LMTO compared to PS-LMTO.

The introduction of lower-valent Mn^{2+} in Mn^{3+}-DRX systems, such as LMTO, is well-documented and primarily attributed to O loss during cycling[3]. Our XAS results confirm that NM-LMTO experiences less O loss and consequently shows reduced surface and bulk Mn reduction compared to PS-LMTO.

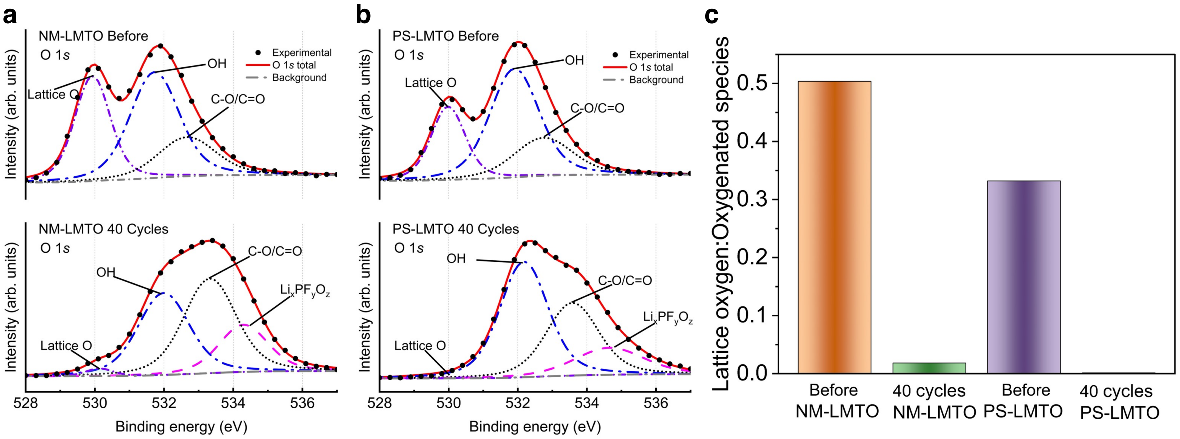

d

O1s peaks	**NM-LMTO before** *Area (CPS.eV)*	**NM-LMTO 40 cycles** *Area (CPS.eV)*	**PS-LMTO before** *Area (CPS.eV)*	**PS-LMTO 40 cycles** *Area (CPS.eV)*
$Li_xPF_yO_z$		30194.84		30035.54
OH	49384.49	54909.14	44302.69	89458.59
C-O/C=O	21409.7	64690.02	18568.11	60994.89
Mn-O-Mn	35650.75	2730.7	20873.85	215.29

Supplementary Figure 20 | XPS analysis of CEI development on NM- and PS-LMTO electrodes. (**a, b**) O 1s XPS spectra of **a** the NM-LMTO electrode, **b** the PS-LMTO electrode before cycling and after 40 cycles in a Li||NM/PS-LMTO cell at 20 mA/g at 25°C within a voltage range of 1.5–4.8 V, and **c** the area ratio of lattice oxygen to oxygenated species in NM-LMTO and PS-LMTO before and after 40 cycles. **d** Areas of oxygen species in NM-LMTO and PS-LMTO before and after 40 Cycles. This table displays the total areas under the O1s peaks for $Li_xPF_yO_z$, OH, C-O/C=O, and Mn-O-Mn, comparing the changes in CEI development between NM-LMTO and PS-LMTO. Before cycling, the as-prepared NM- and PS-LMTO electrodes exhibit an O 1s XPS peak at ~530 eV corresponding to lattice oxygen, along with other oxygen species at higher energies. After 40 cycles, the intensity of the signals from non-lattice oxygen species (OH, C-O/C=O, $Li_xPF_yO_z$) increases, indicating the development of cathode electrolyte interphase (CEI) layers. Notably, in the PS-LMTO electrode, the lattice O signal is completely obscured after 40 cycles, whereas in the NM-LMTO electrode, the lattice O signal remains detectable. This suggests that the NM-LMTO electrode develops thinner CEI layers compared to the PS-LMTO electrode after cycling. Source data for Figs. S20a–S20c are provided as a Source Data file.

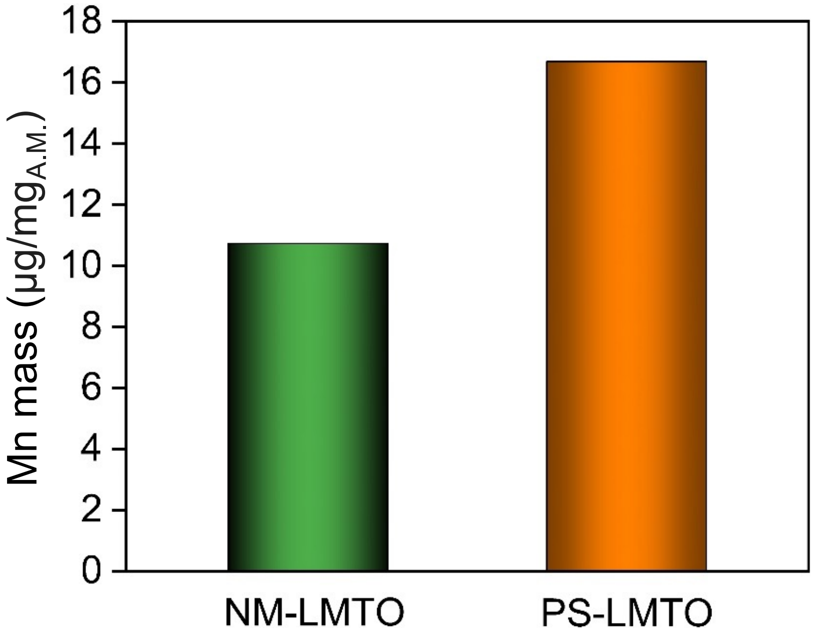

Supplementary Figure 21 | Concentration of Mn deposited on the lithium anode chip, as determined by ICP-OES, after 40 cycles of NM-LMTO and PS-LMTO electrodes at 25°C and 20 mA/g within a voltage range of 1.5–4.8 V in a Li||NM/PS-LMTO cell. The Mn concentration is normalized to the amount of active material (mg of A.M). Source data for Fig. S21 are provided as a Source Data file.

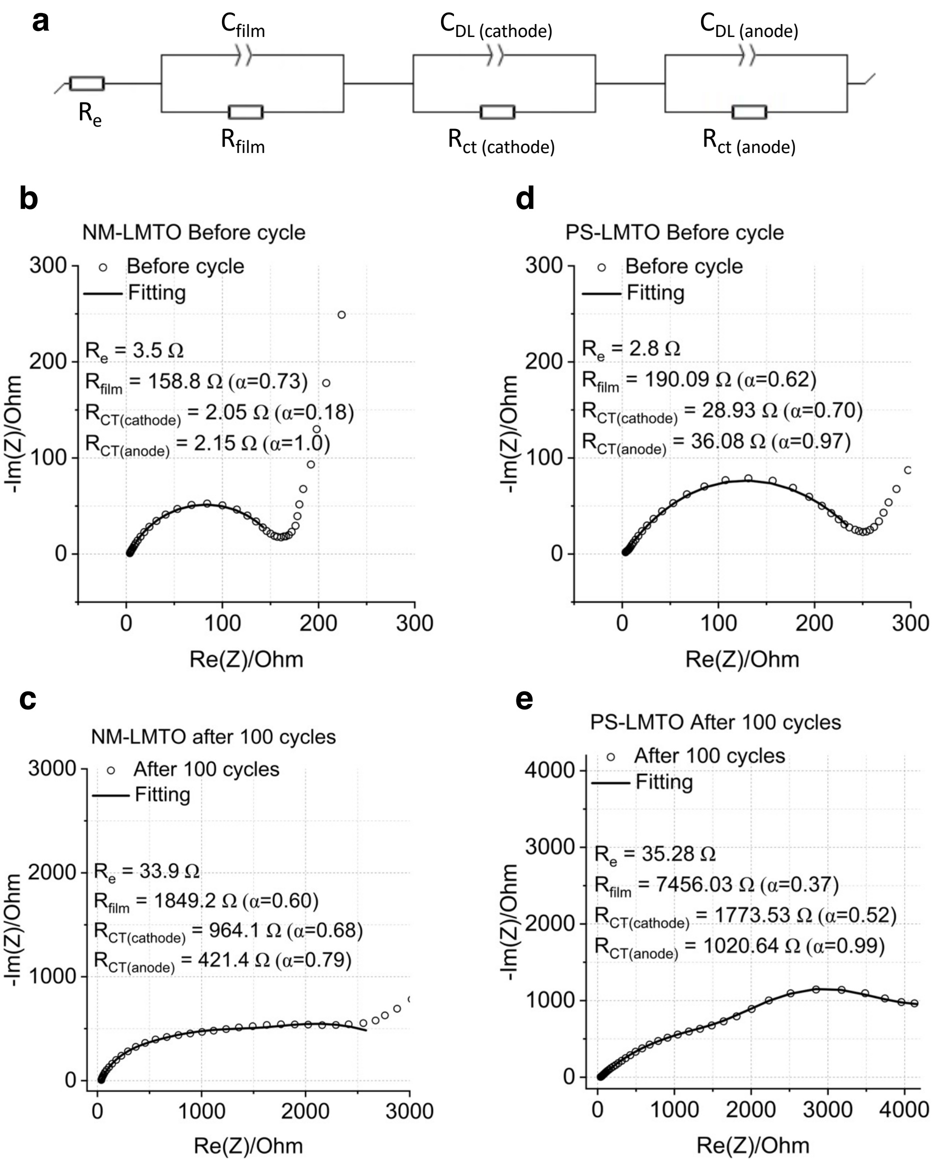

Supplementary Figure 22 | EIS and equivalent circuit fitting of Li||NM/PS-LMTO cells before and after cycling. a The equivalent circuit model used to fit the Nyquist plots of the Li||NM/PS-LMTO cell. We included the electrolyte resistance (R_e), surface film resistance (R_{film}) from the cathode-electrolyte interphase (CEI) or solid-electrolyte interphase (SEI), charge-transfer resistance from the cathode ($R_{ct\ (cathode)}$) and the anode ($R_{ct\ (anode)}$), as well as constant phase elements of C_{film} and double-layer capacitances at the cathode/electrolyte interface ($C_{DL\ (cathode)}$) and the anode/electrolyte interface ($C_{DL\ (anode)}$). (**b–e**) The Nyquist plots of the Li||NM-LMTO cell **b** before cycling and **c** after 100 cycles, and those of the Li||PS-LMTO cell **d** before cycling and **e** after 100 cycles between 1.5–4.8 V at 20 mA/g. It is important to note that a simplified equivalent circuit model, like the one used here, does not conclusively identify the exact source of impedances. Nevertheless, faster impedance growth after cycling is evident for the Li||PS-LMTO cell, as shown by the much larger increases in each resistance component in the equivalent circuit model. Source data for Figs. S22b–S22e are provided as a Source Data file.

a

NM-LMTO before cycle		
Parameter	Value	Standard Error
R_e	3.453 Ω	± 0.04539 Ω
C_{film}	31.81×10^{-6} s^α/Ω	± 0.739×10^{-6} s^α/Ω
α_{film}	0.732	± 2.095×10^{-3}
R_{film}	158.824 Ω	± 0.8617 Ω
$C_{DL\ (cathode)}$	1.4×10^{-5} s^α/Ω	± 0.873×10^{-6} s^α/Ω
$\alpha_{cathode}$	0.17836	± 2.397×10^{-3}
$R_{CT(cathode)}$	2.04536 Ω	± 8.382×10^{-3} Ω
$C_{DL(anode)}$	43.43×10^{-6} s^α/Ω	± 1.019×10^{-6} s^α/Ω
α_{anode}	1.00	± 3.744×10^{-3}
$R_{CT\ (anode)}$	2.150 Ω	± 0.01615 Ω
Chi^2/|Z|^2	0.011	

c

PS-LMTO before cycle		
Parameter	Value	Standard Error
R_e	2.758 Ω	± 0.142 Ω
C_{film}	95.42×10^{-6} s^α/Ω	± 6.78×10^{-6} s^α/Ω
α_{film}	0.623	± 9.4×10^{-4}
R_{film}	190.091 Ω	± 8.7×10^{-1} Ω
$C_{DL\ (cathode)}$	4.3×10^{-4} s^α/Ω	± 2.8×10^{-5} s^α/Ω
$\alpha_{cathode}$	0.703	± 0.010
$R_{CT(cathode)}$	28.926 Ω	± 0.657 Ω
$C_{DL(anode)}$	4.6×10^{-5} s^α/Ω	± 4.6×10^{-6} s^α/Ω
α_{anode}	0.969	± 0.0146
$R_{CT\ (anode)}$	36.081 Ω	± 0.881 Ω
Chi^2/|Z|^2	0.0866	

b

NM-LMTO after 100 cycles		
Parameter	Value	Standard Error
R_e	33.971 Ω	± 1.049 Ω
C_{film}	4.4×10^{-4} s^α/Ω	± 87.99×10^{-6} s^α/Ω
α_{film}	0.730	± 0.029
R_{film}	1849.224 Ω	± 166.659 Ω
$C_{DL\ (cathode)}$	63.722 s^α/Ω	± 13.98×10^{-6} s^α/Ω
$\alpha_{cathode}$	0.683	± 0.037
$R_{CT(cathode)}$	964.102 Ω	± 141.558 Ω
$C_{DL(anode)}$	14.577×10^{-6} s^α/Ω	± 1.909×10^{-6} s^α/Ω
α_{anode}	0.7923	± 0.04522
$R_{CT\ (anode)}$	421.413 Ω	± 121.355 Ω
Chi^2/|Z|^2	0.0068	

d

PS-LMTO after 100 cycles		
Parameter	Value	Standard Error
R_e	35.277 Ω	± 0.599 Ω
C_{film}	1.8×10^{-3} s^α/Ω	± 3.5×10^{-4} s^α/Ω
α_{film}	0.366	± 1.7×10^{-2}
R_{film}	7456.0289 Ω	± 24.2840791 Ω
$C_{DL\ (cathode)}$	2.7×10^{-4} s^α/Ω	± 8.797×10^{-6} s^α/Ω
$\alpha_{cathode}$	0.52118	± 8.5×10^{-3}
$R_{CT(cathode)}$	1773.53 Ω	± 126.965 Ω
$C_{DL(anode)}$	4.7×10^{-3} s^α/Ω	± 5.1×10^{-4} s^α/Ω
α_{anode}	0.9985	± 0.0223
$R_{CT\ (anode)}$	1020.639 Ω	± 115.686 Ω
Chi^2/|Z|^2	0.013	

Supplementary Table 1 | Fitted electrochemical impedance spectroscopy (EIS) parameters with standard errors corresponding to the Nyquist plots shown in Supplementary Fig. 22. Tables **a**, **b**, **c**, and **d** correspond to Supplementary Fig. 22b, 22c, 22d, and 22e, respectively.

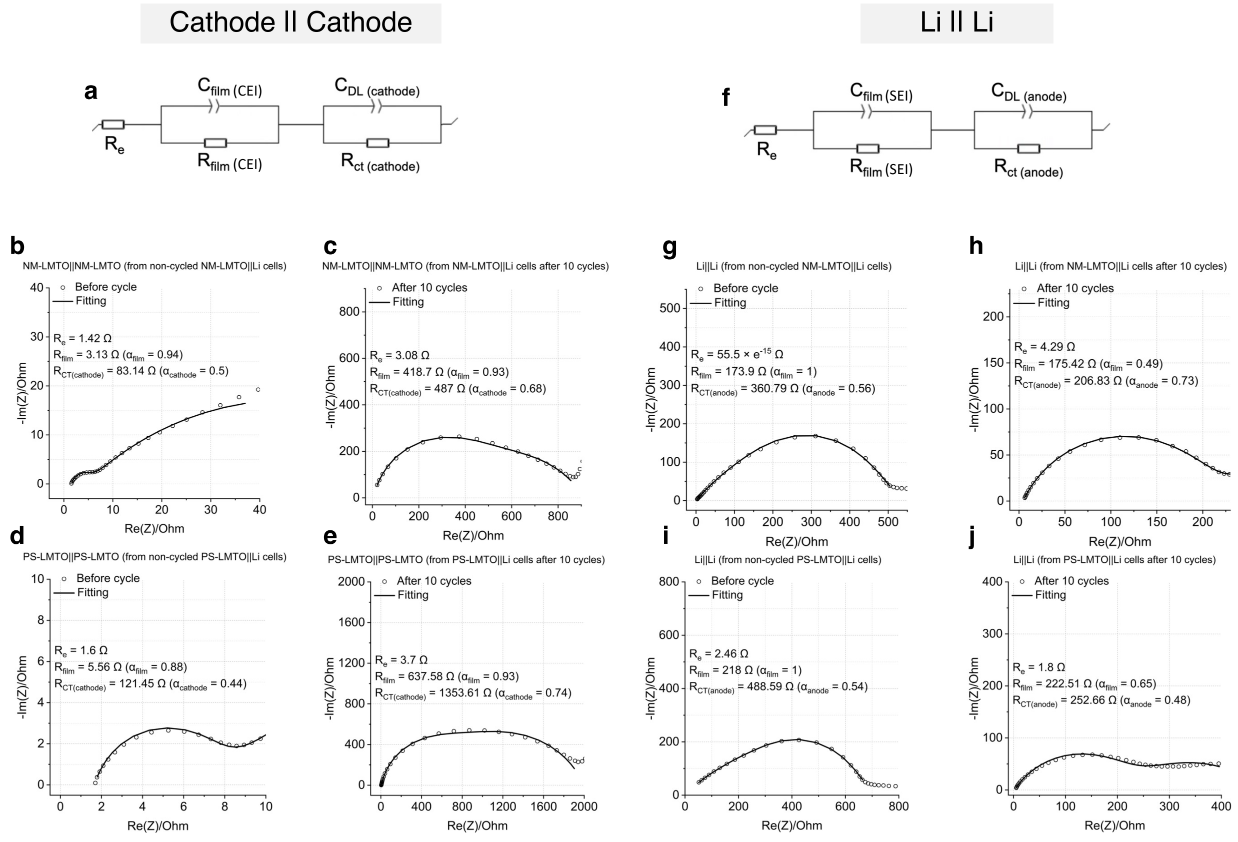

Supplementary Figure 23 | EIS of symmetric cells from NM/PS-LMTO and Li metal electrodes. (**a, f**) The equivalent circuits model used to fit the Nyquist plots of the NM/PS-LMTO||NM/PS-LMTO cathode symmetric cells and Li||Li anode symmetric cells, respectively. (**b–e**) Nyquist plots for symmetric cells assembled with NM- and PS-LMTO cathodes extracted from Li||NM-LMTO and Li||PS-LMTO cells (**b, d**) before cycling and (**c, e**) after 10 cycles between 1.5–4.8 V at 20 mA/g. (**g–j**) Nyquist plots for symmetric cells assembled with Li-metal anodes extracted from Li||NM-LMTO and Li||PS-LMTO cells (**g, i**) before cycling and (**h, j**) after 10 cycles between 1.5–4.8 V at 20 mA/g. Source data for Figs. S23b–S23e and S23g–S23j are provided as a Source Data file.

a

NM-LMTO‖NM-LMTO cathode (Before cycle)		
Parameter	Value	Standard Error
R_e	1.422 Ω	± 0.105 Ω
C_{film}	9.658×10^{-6} s^α/Ω	± 14.124×10^{-6} s^α/Ω
α_{film}	0.939	± 0.061
R_{film}	3.130 Ω	± 0.093 Ω
$C_{DL\ (cathode)}$	0.003 s^α/Ω	± 0.0003 s^α/Ω
$\alpha_{cathode}$	0.498	± 0.019
$R_{CT(cathode)}$	83.141 Ω	± 0.974 Ω
Chi^2/|Z|^2	0.063	

b

NM-LMTO‖NM-LMTO cathode (After 10 cycles)		
Parameter	Value	Standard Error
R_e	3.08 Ω	± 0.0476 Ω
C_{film}	2.735×10^{-3} s^α/Ω	± 0.136×10^{-3} s^α/Ω
α_{film}	0.93	± 0.01996
R_{film}	418.7 Ω	± 14.73 Ω
$C_{DL\ (cathode)}$	4.904×10^{-3} s^α/Ω	± 0.778×10^{-3} s^α/Ω
$\alpha_{cathode}$	0.68	± 0.0702
$R_{CT(cathode)}$	487 Ω	± 0.5829 Ω
Chi^2/|Z|^2	0.021	

c

PS-LMTO‖PS-LMTO cathode (Before cycle)		
Parameter	Value	Standard Error
R_e	1.601 Ω	± 0.108 Ω
C_{film}	13.402×10^{-6} s^α/Ω	± 4.302×10^{-6} s^α/Ω
α_{film}	0.885	± 0.0305
R_{film}	5.565 Ω	± 0.333 Ω
$C_{DL\ (cathode)}$	0.013 s^α/Ω	± 0.002 s^α/Ω
$\alpha_{cathode}$	0.436	± 0.036
$R_{CT(cathode)}$	121.447 Ω	± 3.448 Ω
Chi^2/|Z|^2	0.052	

d

PS-LMTO‖PS-LMTO cathode (After 10 cycles)		
Parameter	Value	Standard Error
R_e	3.697 Ω	± 0.091 Ω
C_{film}	8.116×10^{-6} s^α/Ω	± 0.683×10^{-6} s^α/Ω
α_{film}	0.927	± 0.021
R_{film}	637.577 Ω	± 71.498 Ω
$C_{DL\ (cathode)}$	63.653×10^{-6} s^α/Ω	± 6.407×10^{-6}s^α/Ω
$\alpha_{cathode}$	0.745	± 0.015
$R_{CT(cathode)}$	1353.615 Ω	± 73.679 Ω
Chi^2/|Z|^2	0.034	

e

Li‖Li-anode in the NM-LMTO half cell (Before cycle)		
Parameter	Value	Standard Error
R_e	55.599×10^{-15} Ω	± 0.479×10^{-9} Ω
C_{film}	12.559×10^{-6} s^α/Ω	± 0.360×10^{-6} s^α/Ω
α_{film}	1	± 0
R_{film}	173.939 Ω	± 6.312 Ω
$C_{DL(anode)}$	67.308×10^{-3} s^α/Ω	± 3.064×10^{-6} s^α/Ω
α_{anode}	0.561	± 0.004
$R_{CT\ (anode)}$	360.798 Ω	± 9.801 Ω
Chi^2/|Z|^2	0.021	

f

Li‖Li-anode in the NM-LMTO half cell (After 10 cycles)		
Parameter	Value	Standard Error
R_e	4.289 Ω	± 0.290 Ω
C_{film}	0.003 s^α/Ω	± 0.00013 s^α/Ω
α_{film}	0.494	± 0.039
R_{film}	175.422 Ω	± 17.963 Ω
$C_{DL(anode)}$	8.533×10^{-6} s^α/Ω	± 0.395×10^{-6} s^α/Ω
α_{anode}	0.730	± 0.007
$R_{CT\ (anode)}$	206.830 Ω	± 5.134 Ω
Chi^2/|Z|^2	0.008	

g

Li‖Li-anode in the PS-LMTO half cell (Before cycle)		
Parameter	Value	Standard Error
R_e	2.457 Ω	± 1.610 Ω
C_{film}	12.179×10^{-6} s^α/Ω	± 0.289×10^{-6} s^α/Ω
α_{film}	0.999	± 0.0046
R_{film}	218.0017 Ω	± 5.248 Ω
$C_{DL(anode)}$	44.524×10^{-6} s^α/Ω	± 2.277×10^{-6} s^α/Ω
α_{anode}	0.539	± 0.007
$R_{CT\ (anode)}$	488.58 Ω	± 8.183 Ω
Chi^2/|Z|^2	0.0009	

h

Li‖Li-anode in the PS-LMTO half cell (After 10 cycles)		
Parameter	Value	Standard Error
R_e	1.7978 Ω	± 0.748 Ω
C_{film}	22.060×10^{-6} s^α/Ω	± 3.1072×10^{-6} s^α/Ω
α_{film}	0.645	± 0.0212
R_{film}	222.507 Ω	± 18.171 Ω
$C_{DL(anode)}$	0.0018 s^α/Ω	± 0.00017 s^α/Ω
α_{anode}	0.476	± 0.061
$R_{CT\ (anode)}$	252.664 Ω	± 41.396 Ω
Chi^2/|Z|^2	0.059	

Supplementary Table 2 | Fitted electrochemical impedance spectroscopy (EIS) parameters with standard errors corresponding to the Nyquist plots shown in Supplementary Fig. 23. Tables **a**, **b**, **c**, **d**, **e**, **f**, **g**, and **h** correspond to Supplementary Fig. 23b, 23c, 23d, 23e, 23g, 23h, 23i, and 23j, respectively.

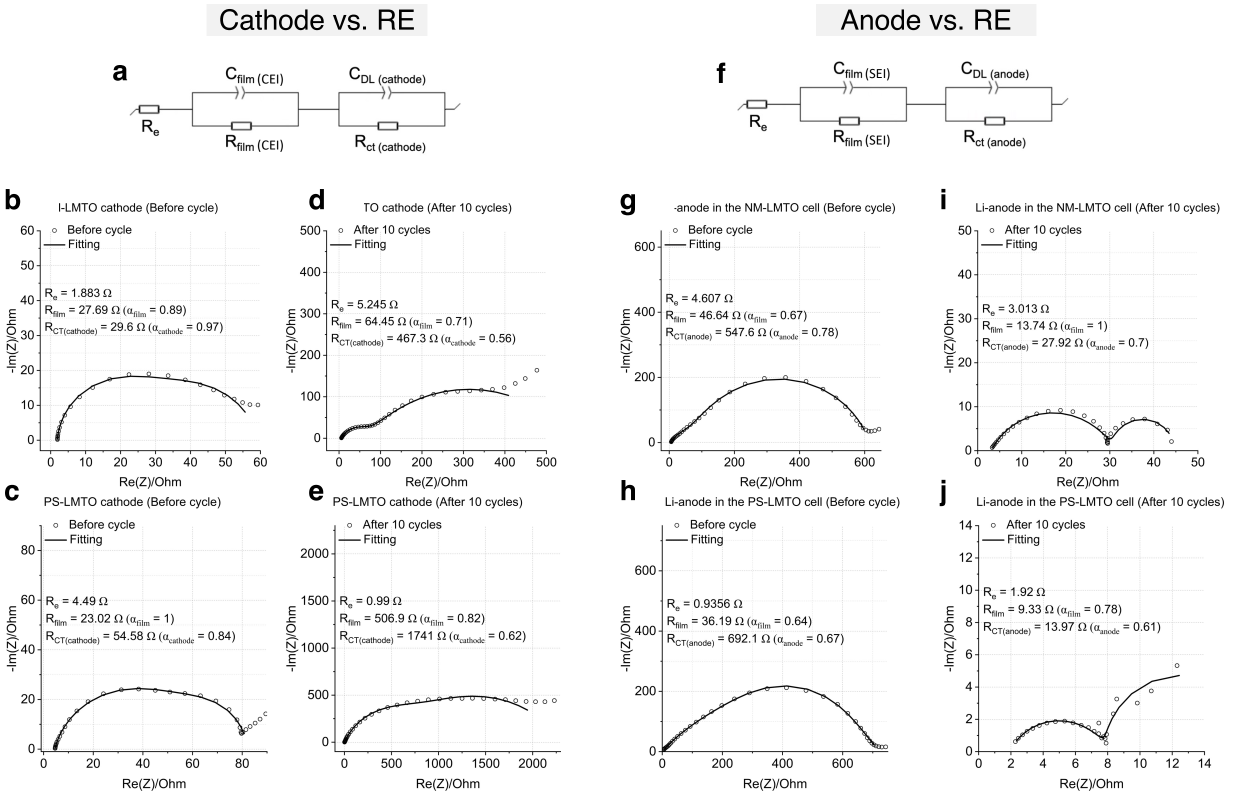

Supplementary Figure 24 | Three-electrode EIS measurements to decouple cathode and anode impedance growth. (**a, f**) Equivalent circuit models used to fit the Nyquist plots measured **a** between the cathode (NM/PS-LMTO electrode) and the reference electrode (Li-deposited Cu mesh), and **f** between the Li-metal anode and the reference electrode, in three-electrode EIS measurements. (**b–e**) Nyquist plots and corresponding fits measured between the cathode and the reference electrode: (**b, c**) before cycling and (**d, e**) after 10 cycles between 1.5–4.8 V at 20 mA/g in the three-electrode cells. (**g–j**) Nyquist plots and corresponding fits measured between the Li-metal anode and the reference electrode: (**g, h**) before cycling and (**i, j**) after 10 cycles under the same conditions. Figs. S24b–S24e and S24g–S24j are provided as a Source Data file.

a

NM-LMTO cathode (Before cycle)		
Parameter	Value	Standard Error
R_e	1.883 Ω	± 4.43×10^{-3} Ω
C_{film}	12.35×10^{-6} s^α/Ω	± 10.8764×10^{-6} s^α/Ω
α_{film}	0.89	± 0.01568
R_{film}	27.69 Ω	± 2.135 Ω
$C_{DL\ (cathode)}$	12.35×10^{-6} s^α/Ω	± 1.919×10^{-6} s^α/Ω
$\alpha_{cathode}$	0.97	± 5.34×10^{-3}
$R_{CT(cathode)}$	29.6 Ω	± 0.08338 Ω
$\mathrm{Chi}^2/\lvert Z\rvert^2$	0.3399	

b

NM-LMTO cathode (After 10 cycles)		
Parameter	Value	Standard Error
R_e	5.245Ω	± 0.0316 Ω
C_{film}	7.945×10^{-6} s^α/Ω	± 0.451×10^{-6} s^α/Ω
α_{film}	0.71	± 5.19×10^{-6}
R_{film}	64.45 Ω	± 0.0462 Ω
$C_{DL\ (cathode)}$	62.02×10^{-6} s^α/Ω	± 2.097×10^{-6} s^α/Ω
$\alpha_{cathode}$	0.56	± 4.89×10^{-3}
$R_{CT(cathode)}$	467.3 Ω	± 20.1 Ω
$\mathrm{Chi}^2/\lvert Z\rvert^2$	0.092	

c

PS-LMTO cathode (Before cycle)		
Parameter	Value	Standard Error
R_e	4.49 Ω	± 0.0588 Ω
C_{film}	13.39×10^{-6} s^α/Ω	± 0.898×10^{-6} s^α/Ω
α_{film}	1	± 6.925×10^{-3}
R_{film}	23.02 Ω	± 1.525 Ω
$C_{DL\ (cathode)}$	65.92×10^{-6} s^α/Ω	± 7.738×10^{-6} s^α/Ω
$\alpha_{cathode}$	0.84	± 7.596×10^{-3}
$R_{CT(cathode)}$	54.58 Ω	± 1.164 Ω
$\mathrm{Chi}^2/\lvert Z\rvert^2$	0.1709	

d

PS-LMTO cathode (After 10 cycles)		
Parameter	Value	Standard Error
R_e	0.99 Ω	± 0.0641 Ω
C_{film}	33.3×10^{-6} s^α/Ω	± 1.821×10^{-6} s^α/Ω
α_{film}	0.82	± 5.568×10^{-3}
R_{film}	506.9 Ω	± 0.0866 Ω
$C_{DL\ (cathode)}$	33.3×10^{-6} s^α/Ω	± 1.758×10^{-6} s^α/Ω
$\alpha_{cathode}$	0.62	± 4.818×10^{-3}
$R_{CT(cathode)}$	1741 Ω	± 66.32 Ω
$\mathrm{Chi}^2/\lvert Z\rvert^2$	0.368	

e

Li-anode in the NM-LMTO cell (Before cycle)		
Parameter	Value	Standard Error
R_e	4.607 Ω	± 0.032 Ω
C_{film}	38.57×10^{-6} s^α/Ω	± 1.372×10^{-6} s^α/Ω
α_{film}	0.67	± 6.859×10^{-6}
R_{film}	46.64 Ω	± 6.957 Ω
$C_{DL(anode)}$	31.31×10^{-6} s^α/Ω	± 4.9×10^{-6} s^α/Ω
α_{anode}	0.78	± 0.00141
$R_{CT\ (anode)}$	547.6 Ω	± 0.2387 Ω
$\mathrm{Chi}^2/\lvert Z\rvert^2$	0.2076	

f

Li-anode in the NM-LMTO cell (After 10 cycles)		
Parameter	Value	Standard Error
R_e	3.013 Ω	± 0.05476 Ω
C_{film}	0.06376 s^α/Ω	± 0.02704 s^α/Ω
α_{film}	1	± 0.02575
R_{film}	13.74 Ω	± 0.2569 Ω
$C_{DL(anode)}$	40.36×10^{-6} s^α/Ω	± 7.87×10^{-6} s^α/Ω
α_{anode}	0.7	± 0.01671
$R_{CT\ (anode)}$	27.92 Ω	± 0.52469 Ω
$\mathrm{Chi}^2/\lvert Z\rvert^2$	0.055	

g

Li-anode in the PS-LMTO cell (Before cycle)		
Parameter	Value	Standard Error
R_e	0.9356 Ω	± 0.3377×10^{-6} Ω
C_{film}	63.31×10^{-6} s^α/Ω	± 1.5×10^{-6} s^α/Ω
α_{film}	0.64	± 3.379×10^{-3}
R_{film}	36.19 Ω	± 1.878 Ω
$C_{DL(anode)}$	25.48×10^{-6} s^α/Ω	± 0.954×10^{-6} s^α/Ω
α_{anode}	0.67	± 4.042×10^{-3}
$R_{CT\ (anode)}$	692.1 Ω	± 12.46 Ω
$\mathrm{Chi}^2/\lvert Z\rvert^2$	0.1286	

h

Li-anode in the PS-LMTO cell (After 10 cycles)		
Parameter	Value	Standard Error
R_e	1.92 Ω	± 0.04692 Ω
C_{film}	41.33×10^{-6} s^α/Ω	± 6.79×10^{-6} s^α/Ω
α_{film}	0.78	± 0.04086
R_{film}	9.33 Ω	± 0.1483 Ω
$C_{DL(anode)}$	41.33×10^{-6} s^α/Ω	± 7.82×10^{-6} s^α/Ω
α_{anode}	0.61	± 0.0161
$R_{CT\ (anode)}$	13.97 Ω	± 0.1678 Ω
$\mathrm{Chi}^2/\lvert Z\rvert^2$	0.03949	

Supplementary Table 3 | Fitted electrochemical impedance spectroscopy (EIS) parameters with standard errors corresponding to the Nyquist plots shown in Supplementary Fig. 24. Tables **a**, **b**, **c**, **d**, **e**, **f**, **g**, and **h** correspond to Supplementary Fig. 24b, 24d, 24c, 24e, 24g, 24i, 24h, and 24j, respectively.

Supplementary Note 3 | Cell impedance analysis

To isolate the impedance growth at the cathode from the Li||NM-LMTO and Li||PS-LMTO cells, we first conducted symmetric cell analyses using electrodes from cycled Li||PS-LMTO and Li||NM-LMTO cells. Our approach involved fabricating two symmetric cells for each material: an anode symmetric cell (Li||Li) and a cathode symmetric cell (Cathode||Cathode). We first measured the initial impedance of these cells before cycling. Then, we disassembled the symmetric cells in an argon-filled glove box and reassembled them as half-cells by pairing each electrode (lithium or cathode) with a fresh counterpart. These half-cells were cycled for 10 cycles, after which they were disassembled again, and the electrodes were swapped back to reconstruct the symmetric cells. Finally, we remeasured the impedance to evaluate changes in each electrode's contribution after cycling.

Supplementary Fig. 23 and Supplementary Table 2 present the Nyquist plots of the Cathode||Cathode and Li||Li symmetric cells. After 10 cycles in the Li||PS/NM-LMTO cells, the total resistance (R_e + R_{film} + R_{ct}) of the PS-LMTO||PS-LMTO cell increased ~15.5-fold (from 128.61 Ω to 1994.89 Ω), whereas in the NM-LMTO||NM-LMTO cell, it increased ~10.4-fold (from 87.69 Ω to 908.78 Ω). Since these are symmetric cells, the observed impedances can be fully attributed to the PS- or NM-LMTO cathode (with impedance doubling due to the identical electrodes). The lower initial impedance in the NM-LMTO symmetric cell and its smaller increase after 10 cycles support our conclusion that the NM-LMTO electrode exhibits greater stability against impedance growth than the PS-LMTO electrode. For the Li||Li symmetric cells derived from the Li||PS-LMTO and Li||NM-LMTO cells, impedance decreased after 10 cycles, likely due to the electrochemical decomposition of the passivation layer on the Li chips.

To further substantiate this observation, we performed three-electrode EIS measurements on NM-LMTO and PS-LMTO coin cells. In these experiments, cathodes composed of 70 wt% NM/PS-LMTO, 20 wt% MWCNT, and 10 wt% PVDF were used as the working electrodes, with a Li-deposited Cu mesh as the reference electrode (RE) and a Li-metal disk as the counter electrode (CE). The three-electrode coin cells were assembled following the protocol reported by Cheuh et al.[4], as also detailed in the Methods section of the manuscript.

Supplementary Fig. 24 and Supplementary Table 3 show the results of the three-electrode EIS measurements. Nyquist plots measured between the cathode and the reference electrode (RE) indicate that initial cathode impedance is lower for the NM-LMTO electrode ($R_{film}+R_{ct(cathode)}$ ≈ 57.3 Ω) compared to the PS-LMTO electrode ($R_{film}+R_{ct(cathode)}$ ≈ 77.6 Ω) (Supplementary Fig. 24b and 24c). Notably, after 10 cycles between 1.5–4.8 V at 20 mA/g, the total resistance increases substantially to ≈ 531.7 Ω for the NM-LMTO cathode and ≈ 2247.9 Ω for the PS-LMTO cathode (Supplementary Fig. 24d and 24e), highlighting a much more pronounced impedance growth for the PS-LMTO cathode.

For the Li-metal anode, the impedance measured against the RE decreases after 10 cycles in both Li||NM-LMTO and Li||PS-LMTO cells (Supplementary Fig. 24i and 24j), likely due to the electrochemical decomposition of the passivation layer on the Li-metal surface. This trend is consistent with the Li||Li symmetric cell EIS results in Supplementary Figure 23. Notably, this does not contradict the impedance increase observed at the Li-metal anode side in Supplementary Figure 22, as the symmetric and three-electrode tests reflect only 10 cycles, whereas Supplementary Figure 22 presents data after 100 cycles. Over extended cycling, Li dendrite formation and surface degradation can substantially increase anode impedance, which is captured in the equivalent circuit fitting shown in Supplementary Figure 22.

These three-electrode EIS results corroborate the observations from symmetric cells and Li||PS/NM-LMTO cells, confirming that the faster capacity fade in Li||PS-LMTO cells primarily stems from significantly accelerated degradation of the PS-LMTO cathode compared to NM-LMTO in Li||NM-LMTO cells. Nonetheless, degradation of the Li-metal anode remains a contributing factor, particularly over long-term cycling.

Supplementary References

1 Lee, J. *et al.* Reversible Mn^{2+}/Mn^{4+} double redox in lithium-excess cathode materials. *Nature* **556**, 185-190 (2018). https://doi.org/10.1038/s41586-018-0015-4

2 Yabuuchi, N. *et al.* High-capacity electrode materials for rechargeable lithium batteries: Li_3NbO_4-based system with cation-disordered rocksalt structure. *Proceedings of the National Academy of Sciences* **112**, 7650-7655 (2015). https://doi.org/doi:10.1073/pnas.1504901112

3 Chen, D., Kan, W. H. & Chen, G. Understanding Performance Degradation in Cation-Disordered Rock-Salt Oxide Cathodes. *Advanced Energy Materials* **9**, 1901255 (2019). https://doi.org/https://doi.org/10.1002/aenm.201901255

4 Kuo, J. J., Kang, S. D. & Chueh, W. C. Contact Resistance of Carbon–Li(Ni,Mn,Co)O_2 Interfaces. *Advanced Energy Materials* **12**, 2201114 (2022). https://doi.org/https://doi.org/10.1002/aenm.202201114
